# PRMT5 Inhibitor Synergizes with Chemotherapy to Induce Resembling Mismatch Repair Deficiency and Enhance Anti‐TIGIT Therapy in Microsatellite‐Stable Colorectal Cancer

**DOI:** 10.1002/advs.202500271

**Published:** 2025-05-08

**Authors:** Jiang Zhu, Shenao Fu, Xi Zou, Hanjiang Zeng, Guangzu Cui, Yinghui Peng, Diya Tang, Fan Zhang, Hong Shen, Shan Zeng, Ying Han

**Affiliations:** ^1^ Department of Oncology Xiangya Hospital 87 Xiangya Road, Kaifu District, Central South University Changsha Hunan 410008 P. R. China; ^2^ National Clinical Research Center for Geriatric Disorders Xiangya Hospital 87 Xiangya Road, Kaifu District, Central South University Changsha Hunan 410008 P. R. China; ^3^ Key Laboratory for Molecular Radiation Oncology of Hunan Province Xiangya Hospital Central South University Changsha Hunan 410008 P. R. China; ^4^ Department of Nephrology Xingsha Campus Hunan Provincial People's Hospital Changsha Hunan 410100 P. R. China; ^5^ College of Life Science Mudanjiang Medical University Mudanjiang 157011 P. R. China

**Keywords:** anti‐tumor immunity, cGAS–STING signaling, colorectal cancer, epigenetic therapies, PRMT5

## Abstract

Microsatellite stable (MSS) colorectal cancer (CRC) is considered an “immune‐cold” tumor, accounting for ≈85% of all CRC cases. The overall response rate to chemotherapy combined with immune checkpoint inhibitors in MSS CRC is typically less than 10%. The specific mechanism that enhances chemotherapy sensitivity and mediated immunogenicity renders MSS CRC more responsive to immunotherapy remains elusive. Experiments in this study identify a DNA damage repair‐related epigenetic gene, protein arginine methyltransferase 5 (PRMT5), whose inhibition enhances Irinotecan (CPT‐11) sensitivity and synergistically induces a postmeiotic segregation increased 2 (PMS2)‐deficient‐like state, leading to the release of cytosolic double‐stranded DNA. This activates the cyclic GMP‐AMP synthase (cGAS)‐stimulator of the IFN gene (STING) signaling pathway, thereby enhancing anti‐tumor immunotherapy through dendritic cell‐T cell‐dependent functions. Importantly, combining the epigenetic anti‐tumor drug GSK3326595 with CPT‐11 significantly upregulates the immune receptor tyrosine‐based inhibitory motif (TIGIT) level on CD8+ T cells and subsequently demonstrates impressive anti‐tumor efficacy in vivo when additional anti‐TIGIT is included. Collectively, this study reveals the crucial role of PRMT5 blockade combined with CPT‐11 in inducing a mismatch repair deficiency‐like state and provides a novel triple‐drug combination therapy strategy as a potential treatment for patients with MSS CRC.

## Graphical abstract

Microsatellite‐stable (MSS) colorectal cancer (CRC) resists immunotherapy. PRMT5 inhibition enhances CPT‐11 sensitivity, inducing a PMS2‐deficient‐like state and cytosolic dsDNA release, which activates the cGAS‐STING pathway to promote anti‐tumor immunity. However, this combination therapy upregulates TIGIT expression on CD8+ T cells, and the addition of anti‐TIGIT significantly enhances therapeutic efficacy, thus providing a novel triple‐drug strategy for MSS CRC.

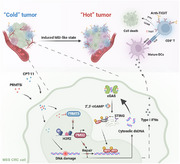

## 1 Introduction

Colorectal cancer (CRC) is the fourth deadliest malignancy worldwide, with an estimated 1.9 million new diagnoses and 0.9 million deaths annually.^[^ ^1^, ^2^ ^]^ Nearly 85% of CRC patients present with the microsatellite stable (MSS) subtype, and the first‐line systemic treatment for MSS CRC typically involves cytotoxic chemotherapy, often with a two‐ or three‐drug regimen (5‐fluorouracil, Oxaliplatin, and CPT‐11).^[^ ^3^, ^4^ ^]^ Emerging evidence suggests that chemotherapy can enhance the efficacy of immune checkpoint inhibitors (ICIs) in solid tumors by activating immunogenicity.^[^ ^5^ ^]^ However, the overall response rate (ORR) of chemotherapy combined with immunotherapy in MSS CRC remains below 10%, as it is characterized by a profoundly immunosuppressive tumor microenvironment (TME), known as a “immune‐cold” tumor.^[^ ^6^, ^7^ ^]^ This highlights the urgent need to investigate the mechanisms underlying the enhancement of chemotherapy sensitivity and immunogenicity within the TME of MSS CRC.

Epigenetics encompasses heritable modifications in gene expression that occur without changes to the underlying DNA sequence, including DNA methylation, histone alterations, and the regulation by non‐coding RNAs.^[^ ^8^ ^]^ Epigenetic therapies for immune modulation remain a significant focus in translational oncology.^[^ ^9^ ^]^ Nonetheless, a recent Phase II single‐arm clinical trial evaluating the combination of the DNMT inhibitors azacitidine and pembrolizumab in MSS CRC reported an ORR of only 3%.^[^ ^10^ ^]^ Moreover, histone deacetylase inhibitors have been reported to enhance the anti‐tumor effects of chemotherapy drugs by modifying the chromatin structure and regulating DNA repair factor expression.^[^ ^11^ ^]^ However, whether targeted epigenetic treatment strategies can enhance chemotherapy‐mediated DNA damage and immunogenicity in MSS CRC remains unclear.

The cyclic GMP‐AMP synthase (cGAS)‐stimulator of the IFN gene (STING) signaling pathway plays a pivotal role in regulating both innate and adaptive immune responses.^[^ ^12^ ^]^ Upon binding to cytosolic DNA, cGAS synthesizes cyclic GMP‐AMP (cGAMP), a second messenger that activates STING.^[^ ^13^ ^]^ This activation initiates a signaling cascade, leading to the production of type I interferons (IFNs) and other immune‐related molecules.^[^ ^14^ ^]^ The cGAS‐STING pathway and its role in anti‐tumor immunity can also be activated by DNA‐damaging drugs.^[^ ^15^ ^]^ The accumulation of DNA damage has been reported to lead to the leakage of damaged DNA into the cytoplasm, which further activates the cGAS‐STING pathway.^[^ ^16^ ^]^ However, activation of the STING pathway by single‐agent chemotherapy is insufficient to induce immunogenic cell death.^[^ ^17^ ^]^ Therefore, enhancing the effectiveness of chemotherapeutic drugs could potentially amplify the STING pathway and enhance anti‐tumor immunity.

Protein arginine methyltransferase 5 (PRMT5), a key enzyme linked to carcinogenesis, mediates the symmetric dimethylation of arginine residues in histone and non‐histone proteins.^[^ ^18^ ^]^ PRMT5 modulates gene expression by generating histone marks, including H3R2me2s, H3R8me2s, and H4R3me2s, and is essential for pre‐mRNA splicing, DNA damage repair (DDR), transcriptional regulation, and cell signaling.^[^ ^19^ ^]^ While PRMT5 inhibition has been reported to downregulate DDR genes and enhance anti‐tumor immune responses, its limited efficacy as a monotherapy has hindered clinical advancement.^[^ ^20^, ^21^ ^]^ The PRMT5 inhibitor (PRMT5i), GSK3326595, is currently in Phase II clinical development for hematological cancers, with combination strategies alongside chemotherapy, targeted therapy, and immunotherapy emerging as pivotal future research directions to enhance therapeutic efficacy.^[^ ^22^ ^]^ However, the role of PRMT5 inhibitors in MSS CRC remains unclear.

In this study, PRMT5 was investigated as a potential DDR‐related epigenetic target in MSS CRC cells using single‐cell RNA sequencing (scRNA‐seq) and bulk RNA sequencing (RNA‐seq). We found that PRMT5 inhibition in combination with the DNA‐damaging chemotherapeutic drug CPT‐11 induced a dMMR‐like state, thereby enhancing cGAS‐STING pathway activation and anti‐tumor immune reprogramming, ultimately increasing the sensitivity to ICIs treatment in MSS CRC. Furthermore, the combination of PRMT5i with CPT‐11 significantly enhanced tumor immunogenicity and strengthened anti‐tumor immunity through dendritic cell (DC)‐T cell‐dependent functions. Overall, our findings revealed a novel mechanism of immune regulation triggered by combination therapy, offering a strategy to mimic the state of dMMR to increase tumor sensitivity to ICIs. This provides an additional mechanistic rationale for the potential application of this novel combination therapy as a promising and effective treatment for MSS CRC.

## 2 Results

### 2.1 PRMT5 as an Epigenetic Target is Associated with DNA Damage Repair in MSS CRC Patients

**Figure 1:**
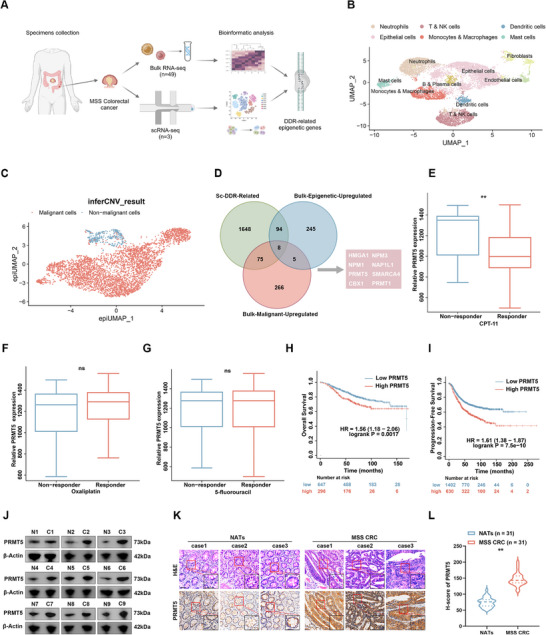
PRMT5 as a DDR‐related epigenetic gene is upregulated in MSS CRC. A) Schematic illustration of the DDR‐related epigenetic gene screening from MSS CRC. The schematic illustration was created using Biorender. B) Uniform manifold approximation and projection (UMAP) plots of single cells identified by scRNA‐seq, color‐coded by major cell types. C) epiUMAP plot representing malignant and non‐malignant cells detected by inferCNV. D) Venn diagram illustrating the intersection of highly expressed epigenetic genes, DDR‐related gene sets in malignant cells, and a malignancy‐promoting gene dataset. E–G) Correlation between PRMT5 levels and the therapeutic effect of DNA‐damaging drugs: CPT‐11 (E), oxaliplatin (F), and 5‐fluorouracil (G), analyzed using ROC plotter (https://rocplot.org/). H,I) Kaplan–Meier survival curves showing OS (H) and PFS (I) for CRC patients with low versus high PRMT5 expression, from the Kaplan–Meier plotter (https://www.kmplot.com/analysis/). J) Western blot analysis of PRMT5 levels in MSS CRC tissues and NATs (*n* = 9). K,L) Representative images (K) and quantitative proportions (L) from IHC staining showing PRMT5 expression in MSS CRC tissues paired with corresponding NATs (*n* = 31). The *p*‐value was calculated using a two‐tailed Student's *t*‐test. Scale bar = 50 µm. Error bars show the mean ± SD. ns, *p* > 0.05, *p < 0.05, **p < 0.01.

To explore the role of epigenetic targets in chemotherapy sensitivity in MSS CRC, highly expressed epigenetic genes were screened from the bulk RNA‐seq data of 49 patients with MSS CRC (Table S1, Supporting Information). Tumor cells often repair chemotherapy‐induced DNA damage through DDR pathways, thereby evading the effects of chemotherapeutic drugs.^[^ ^23^ ^]^ we performed scRNA‐seq on three MSS CRC tissue samples. After annotating the cell types, epithelial cells were isolated and regrouped into malignant and non‐malignant groups based on the inferCNV results (**Figure** **1**A–C; Figure S1A–C, Supporting Information). We then evaluated DDR‐related genes that showed a significant correlation with the DDR status in malignant cells based on ssGSEA analysis of DDR genes identified in a previous study^[^ ^24^ ^]^ (Figure S1D–G, Supporting Information). The intersection of the two gene datasets above with the upregulated malignancy‐related genes, which were screened from bulk RNA‐seq data generated from three pairs of MSS CRC tissues and normal adjacent tissues (NATs), was identified. This analysis revealed eight (*HMGA1, NPM1, PRMT5, CBX1, NPM3, NAP1L1, SMARCA4, PRMT1*) candidate epigenetic genes that are associated with both DDR and malignancy in MSS CRC (Figure 1D). Given the unmet need for improving chemoimmunotherapy in MSS CRC and the established clinical safety of PRMT5 inhibitors, we focused on PRMT5 targeting based on Phase II clinical trials‐proven efficacy of GSK3326595 in both hematologic and solid malignancies.^[^ ^22^ ^]^ However, the potential of PRMT5i in combination with chemotherapeutic agents and its underlying mechanisms in MSS CRC remain unexplored.

The correlation between PRMT5 levels, as a DDR‐related gene, and the response to first‐line DNA‐damaging chemotherapeutic agents, including CPT‐11, oxaliplatin, and 5‐fluorouracil, in patients with CRC was further analyzed. PRMT5 expression was remarkably higher in patients who did not respond to CPT‐11 chemotherapy than in those who responded to treatment (Figure 1E). However, this phenomenon was not observed in CRC patients treated with oxaliplatin or 5‐fluorouracil (Figure 1F,G). Furthermore, Kaplan–Meier analysis demonstrated that higher PRMT5 expression correlated with reduced overall survival (OS) and progression‐free survival (PFS) in CRC patients. (Figure 1H,I). Western blotting and immunohistochemistry (IHC) analyses showed that PRMT5 levels in MSS CRC tissues were significantly higher than those in NATs (Figure 1J–L). Taken together, these results indicate that PRMT5 is associated with DNA damage repair and is upregulated in patients with MSS CRC.

### 2.2 PRMT5 Promotes Cell Viability and Weakens CPT‐11 Sensitivity in MSS CRC Cells

**Figure 2:**
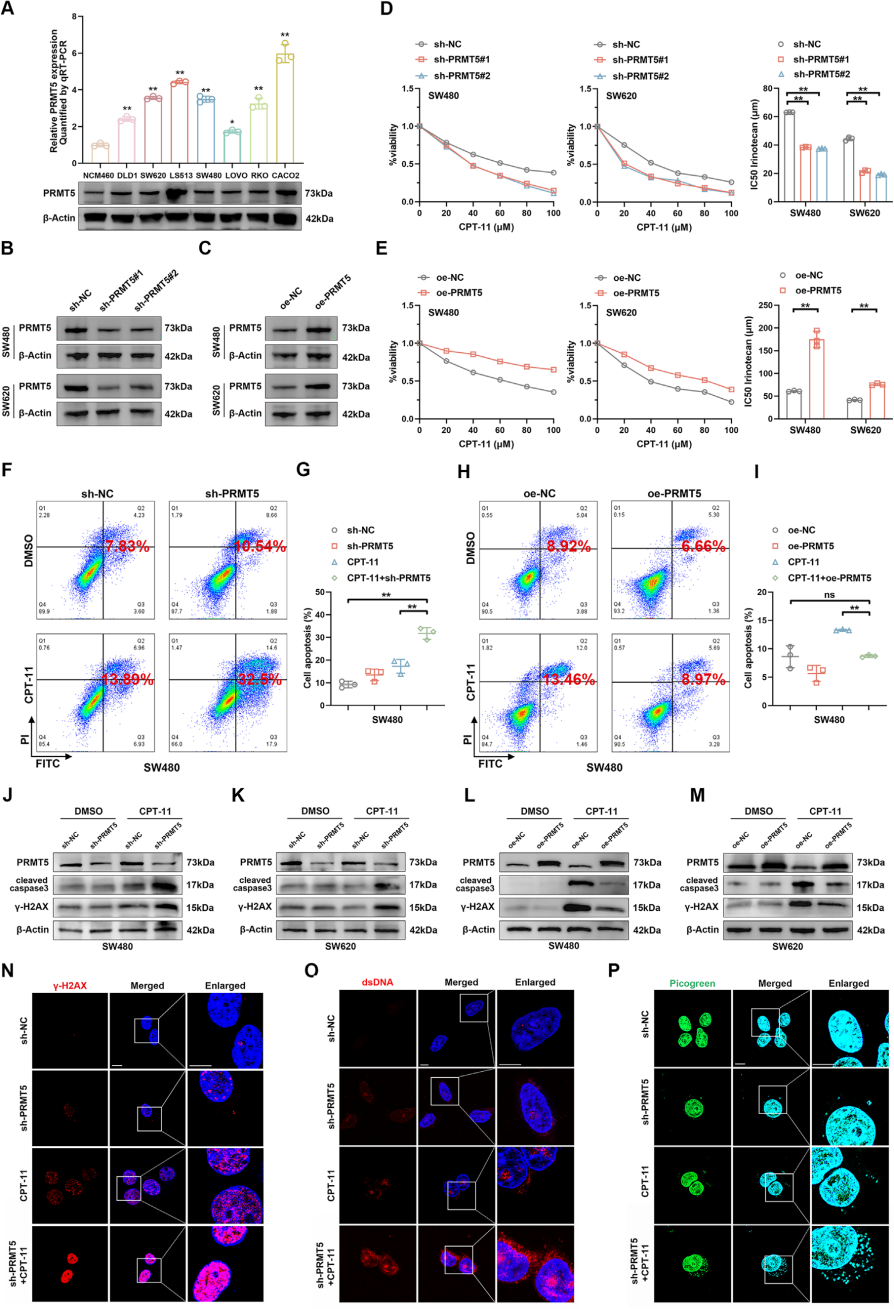
PRMT5 inhibition enhances CPT‐11 sensitivity in vitro. A) qRT‐PCR (top) and Western blot (bottom) analysis of PRMT5 levels in normal human colon mucosal epithelial cells and CRC cell lines. The p‐values were calculated using one‐way ANOVA. B,C) PRMT5 levels were assessed using Western blot in PRMT5 knockdown (B), PRMT5 overexpression (C), and paired control CRC cells. D, E) PRMT5 knockdown (D), PRMT5 overexpression (E), and paired control CRC cells were exposed to increasing concentrations of CPT‐11 for 48 h, and IC50 values were determined using the CCK‐8 assay. The *p*‐values were calculated using one‐way ANOVA. F–I) Flow cytometry analysis of apoptosis in PRMT5 knockdown (F,G), PRMT5 overexpression (H,I), and paired control SW480 cells treated with DMSO or CPT‐11 (50 µm) for 48 h. The *p*‐values were calculated using one‐way ANOVA. J–M) Western blot detection of cleaved caspase‐3 and γ‐H2AX levels in PRMT5 knockdown (J,K), PRMT5 overexpression (L,M), and paired control CRC cells treated with DMSO or CPT‐11 (50 µm). N–P) Representative fluorescence images showing cellular localization and expression of γ‐H2AX (N), dsDNA (O), and Picogreen (P) in PRMT5 knockdown and paired control CRC cells, with or without CPT‐11 (50 µm) treatment. Scale bar = 5 µm. Error bars show the mean ± SD. ns, *p* > 0.05, *p < 0.05, **p < 0.01.

The functional activity of PRMT5 in MSS CRC was further investigated in vitro. Notably, PRMT5 expression was significantly higher in CRC cell lines, including DLD1, SW620, LS513, SW480, LOVO, RKO, and CACO2, compared to normal human colon mucosal epithelial cells (NCM460) (**Figure** **2**A). The SW480 and SW620 cell lines, identified as MSS CRC cell lines^[^ ^25^ ^]^ exhibiting moderate PRMT5 levels, were selected for subsequent functional assays. PRMT5 expression was successfully downregulated by transfection with short hairpin RNAs (shRNAs) and upregulated by transfection with a PRMT5‐overexpressing plasmid (Figure 2B,C). CCK‐8 and colony formation assays demonstrated that PRMT5 knockdown inhibited the proliferation of SW480 and SW620 cells, whereas PRMT5 overexpression had the opposite effect (Figure S2A–H, Supporting Information). Additionally, the 5‐Ethynyl‐2′‐deoxyuridine (EdU) assay confirmed that PRMT5 silencing inhibited DNA replication in CRC cell lines (Figure S2I–P, Supporting Information).

As bioinformatic analyses revealed that PRMT5 expression was higher in patients unresponsive to CPT‐11 chemotherapy, the correlation between PRMT5 expression and CPT‐11 sensitivity in MSS CRC cell lines was evaluated. PRMT5 knockdown significantly increased CPT‐11 sensitivity in SW480 and SW620 cells compared to controls, as indicated by lower half‐maximal inhibitory concentration 50 (IC50) values, higher apoptosis rates, and elevated levels of cleaved caspase‐3. Conversely, cells overexpressing PRMT5 exhibited opposite results (Figure 2D–M; Figure S3A–D, Supporting Information). CPT‐11 exerts cytotoxic effects by inducing DSBs that lead to extensive DNA damage and trigger the release of double‐stranded DNA (dsDNA) from drug‐sensitive CRC cells,^[^ ^26^ ^]^ prompting further investigation into the effects of PRMT5 on CPT‐11 induced DNA damage and dsDNA leakage in CRC cells. Western blot and immunofluorescence (IF) assays revealed that PRMT5 knockdown elevated γ‐H2AX levels in CRC cells following CPT‐11 treatment (Figure 2J–N; Figure S3E, Supporting Information). Similarly, an increased release of dsDNA into the cytoplasm was observed in PRMT5‐silenced CRC cells following treatment with CPT‐11, but not with 5‐fluorouracil or oxaliplatin. (Figure 2O,P; Figure S3F–I, Supporting Information). These findings suggested that PRMT5 abolishes CPT‐11 sensitivity in MSS CRC cells by promoting DNA damage repair.

### 2.3 PRMT5 Inhibitor Combined with CPT‐11 Synergistic Anti‐Tumor of MSS CRC In Vivo

**Figure 3:**
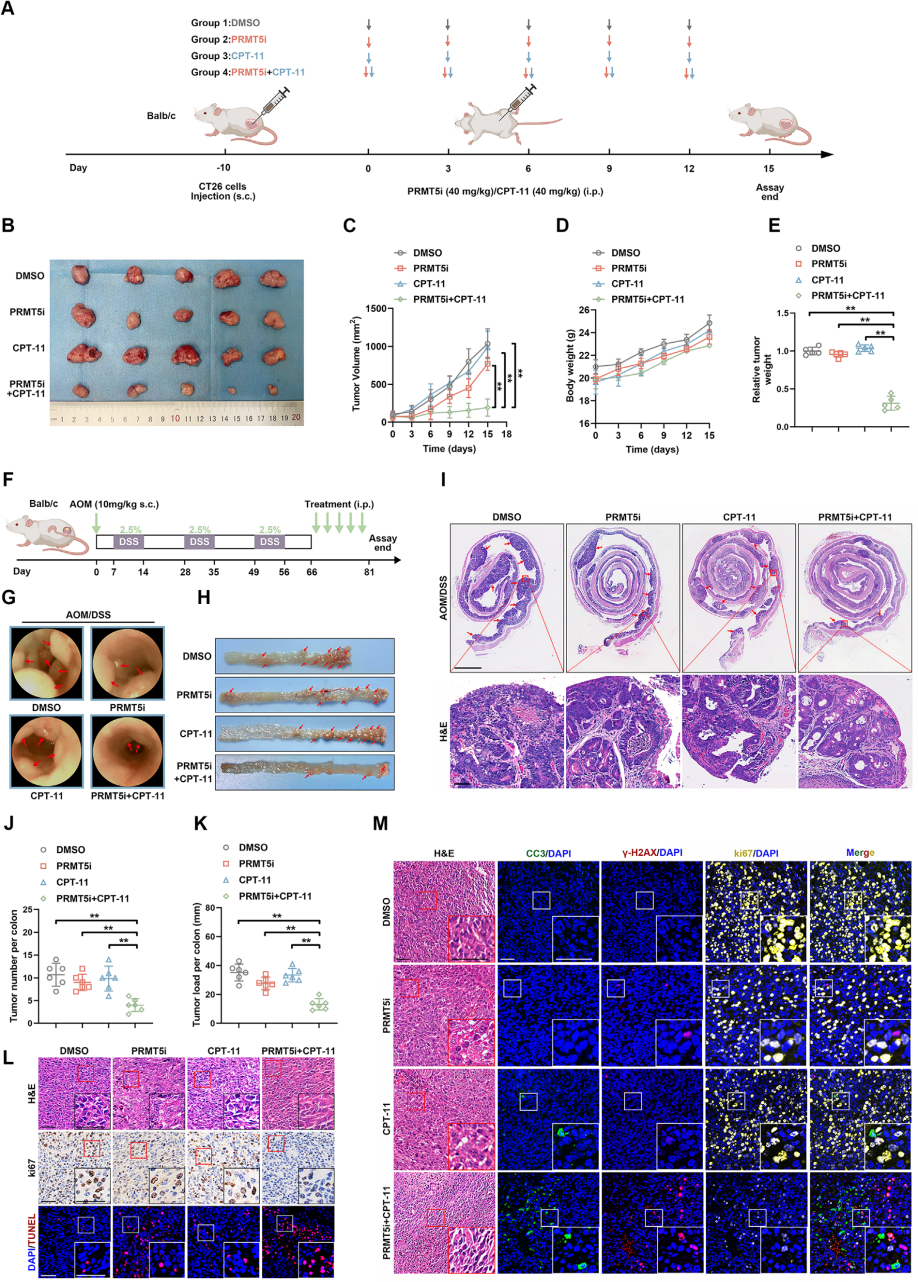
The combination of PRMT5 inhibitor and CPT‐11 impairs tumor growth in MSS mice. A) Schematic timeline for the treatment of mice carrying subcutaneous syngeneic tumors. B–E) Representative tumor images (B), tumor growth curves (C), body weight (D), and relative tumor weight (E) of mice treated with DMSO, PRMT5i (40 mg kg^−1^), CPT‐11 (40 mg kg^−1^), or PRMT5i + CPT‐11 (*n* = 5). The *p*‐values were calculated using two‐way ANOVA (C) and one‐way ANOVA (E). F) Schematic overview of the AOM/DSS model. G–K) Representative mini‐endoscopy images (G), tumor images (H), H&E staining (I), tumor numbers (J), and tumor load (K) in AOM/DSS‐induced tumor‐bearing mice treated with DMSO, PRMT5i (40 mg kg^−1^), CPT‐11 (40 mg kg^−1^), or PRMT5i + CPT‐11 (n = 5). The *p*‐values were calculated using one‐way ANOVA. Scale bar (upper part) = 2 mm, Scale bar (lower part) = 50 µm. L) H&E, ki‐67, and TUNEL staining of tumor tissues across different groups. Scale bar = 50 µm. M) Representative fluorescence images of cleaved caspase‐3, γ‐H2AX, and ki‐67 in the indicated tumor tissues. Scale bar = 50 µm. Error bars show the mean ± SD. ns, *p* > 0.05, *p < 0.05, **p < 0.01.

To further elucidate the role of PRMT5 in CPT‐11 sensitivity in vivo, we established a subcutaneous syngeneic model by injecting CT26 cells into Balb/c mice, which is generally considered equivalent to the MSS state.^[^ ^27^ ^]^ MSS status was confirmed by the positive expression of mismatch repair (MMR) genes in IHC (Figure S4A, Supporting Information). Once the subcutaneous tumor volume reached 200 mm^3^, a combination of the dose‐escalating PRMT5 inhibitors GSK3326595 and CPT‐11 was administered to determine the optimal therapeutic dose (Figure S4B–D, Supporting Information). In vivo assays demonstrated that PRMT5i or CPT‐11 alone had limited effects on tumor suppression, whereas dual treatment significantly reduced tumor volumes (**Figure** **3**A–E). Survival analysis indicated that the dual‐drug combination therapy extended the lifespan of the mice (Figure S4E, Supporting Information). Further combination treatment of PRMT5i with 5‐fluorouracil, oxaliplatin, or CPT‐11 demonstrated that PRMT5i combined with CPT‐11 exhibited the strongest synergistic anti‐tumor effect (Figure S4F–H, Supporting Information). An orthotopic CRC model in Balb/c mice was established using azoxymethane (AOM) and dextran sodium sulfate (DSS) induction (Figure 3F). Consistently, the combination of PRMT5i and CPT‐11 resulted in fewer and smaller tumors than single‐drug treatments (Figure 3G–K). Moreover, IHC and TUNEL staining demonstrated that the combination therapy group, while ensuring safety, exhibited the most potent effects on inhibiting proliferation and promoting apoptosis (Figure 3L,M; Figure S4I–N, Supporting Information). Specifically, dual treatment resulted in the lowest levels of ki67 and the highest levels of cleaved caspase‐3, γ‐H2AX, and TUNEL staining.

### 2.4 PRMT5 Inhibition Combined with CPT‐11 Induces dMMR‐Like CRC

**Figure 4:**
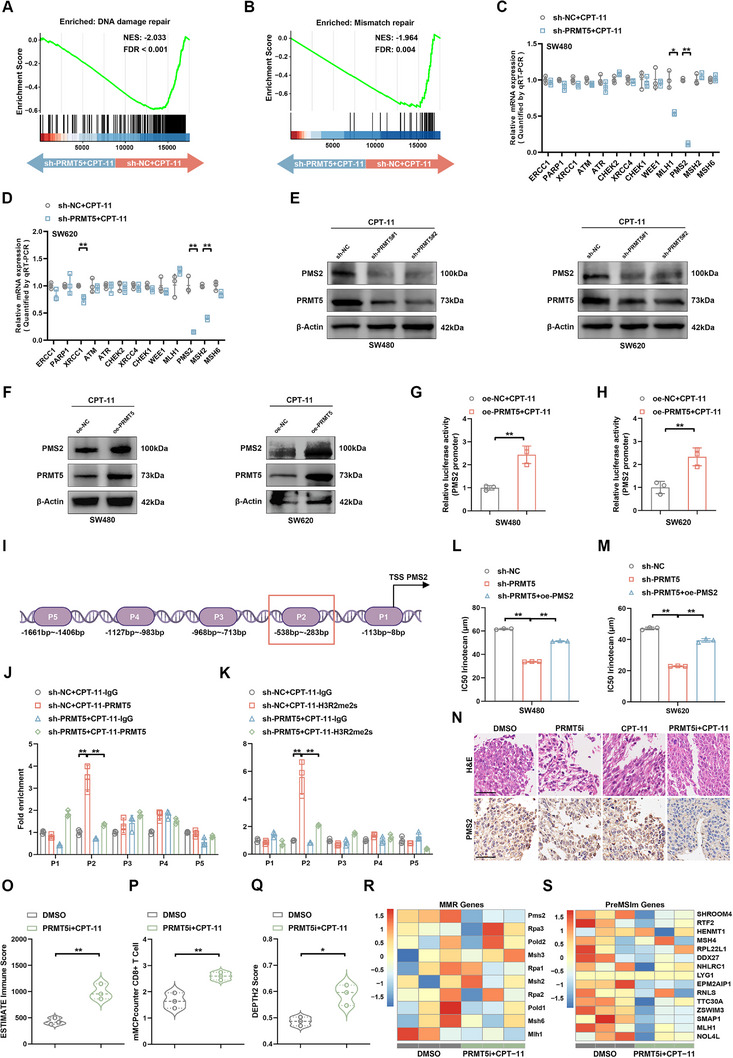
The dual‐drug treatment facilitates the PMS2‐related transformation of dMMR‐like CRC. A,B) Enrichment analysis of DNA damage repair (A) and mismatch repair (B) signaling pathways in SW620 cells from CPT‐11 versus PRMT5 inhition + CPT‐11 groups, conducted using GSEA (*n* = 3). NES, normalized enrichment score; FDR, false discovery rate. C,D) qRT‐PCR analysis of the expression of 13 candidate DDR genes in control and PRMT5‐silenced SW480 (C) and SW620 (D) cells following CPT‐11 treatment. The *p*‐values were calculated using a two‐tailed Student's *t*‐test. E,F) Western blot analysis of PMS2 protein levels in PRMT5 knockdown (E), PRMT5 overexpression (F), and paired control SW480 and SW620 cells treated with CPT‐11. G,H) Luciferase reporter assay showing the effect of PRMT5 on the transcriptional activity of the PMS2 promoter in CPT‐11‐treated SW480 (G) and SW620 (H) cells. The *p*‐values were calculated using a two‐tailed Student's *t*‐test. I) Schematic illustration of putative PRMT5‐binding sites on the PMS2 promoter region and the design of corresponding primers. J,K) ChIP‐qPCR analysis showing the enrichment of PRMT5, H3R2me2s, or IgG at the PMS2 promoter. The *p*‐values were calculated using two‐way ANOVA. L, M) IC50 values of SW480 (L) and SW620 (M) cells assessed using the CCK‐8 assay following 48 h treatment with increasing concentrations of CPT‐11 across different groups. The *p*‐values were calculated using one‐way ANOVA. N) IHC staining showing PMS2 protein levels in mouse tumor tissues from the indicated groups. Scale bar = 50 µm. O) ESTIMATE immune score estimation in mice tumors treated with DMSO versus PRMT5i combined with CPT‐11, based on RNA‐seq data (*n* = 3). The *p*‐values were calculated using a two‐tailed Student's *t*‐test. P) mMCPCounter analysis showing CD8^+^ T cell infiltration in the indicated treatment groups (*n* = 3). The *p*‐values were calculated using a two‐tailed Student's *t*‐test. Q) DEPTH2 scores for mice samples from the indicated treatment groups (*n* = 3). The *p*‐values were calculated using a two‐tailed Student's *t*‐test. R) Heatmap showing the relative expression of MMR‐related genes in the indicated treatment groups (*n* = 3). S) MSI status as determined by PreMSIm in the indicated treatment groups (*n* = 3). MSI‐low (MSI‐L) represents pMMR, and MSI‐high (MSI‐H) represents dMMR. Error bars show the mean ± SD. ns, *p* > 0.05, *p < 0.05, **p < 0.01.

To further investigate the mechanism underlying enhanced cell apoptosis and reduced IC50 of CPT‐11 following PRMT5 suppression, RNA‐seq was performed on CRC cells treated with CPT‐11 alone, as well as on cells treated with both PRMT5 inhibiton and CPT‐11. Multiple DNA‐repair pathways were identified using standardized gene set variation analysis scores.^[^ ^28^ ^]^ Notably, samples with low PRMT5 expression exhibited a significantly reduced DNA repair response to CPT‐11 treatment, suggesting that PRMT5 inhibition broadly influences DNA repair mechanisms (Figure S5A, Supporting Information). Furthermore, there was a positive correlation between PRMT5 expression and the signatures of DDR and MMR genes post‐CPT‐11 treatment, as highlighted by gene set enrichment analysis (GSEA) plots^[^ ^29^ ^]^ (**Figure** **4**A,B). Next, 13 candidate DDR genes, which have been confirmed to potentially bind to DNA damage in CRC, were selected. Among them, only PMS2 levels were positively correlated with PRMT5 levels in both SW480 and SW620 cells exposed to CPT‐11, as revealed by quantitative reverse transcription PCR (qRT‐PCR) (Figure 4C,D; Figure S5B,C, Supporting Information). Consistent with previous reports demonstrating that DNA‐damaging chemotherapy induces transient downregulation of MMR components to promote adaptive mutagenesis and therapeutic resistance,^[^ ^30^ ^]^ our study revealed treatment‐associated modulation of PMS2 expression. Specifically, CPT‐11 monotherapy led to a modest reduction in PMS2 levels, while the combination treatment resulted in a more pronounced decrease (Figure S5D,E, Supporting Information). Western blot analysis consistently showed that PRMT5 knockdown suppressed PMS2 expression, whereas PRMT5 overexpression upregulated PMS2 expression in CPT‐11‐treated CRC cells (Figure 4E,F). Since PRMT5 promotes gene transcription via symmetric methylation of H3R2me2s,^[^ ^19^ ^]^ we explored its potential role in directly modulating PMS2 transcription. A dual‐luciferase reporter system was employed and verified that PRMT5 enhanced the reporter activity driven by the PMS2 promoter following CPT‐11 treatment (Figure 4G,H). To further validate the binding range of PRMT5 to the PMS2 promoter region, we designed five pairs of Chromatin Immunoprecipitation (ChIP) qRT‐PCR primers targeting specific sites within the PMS2 promoter (Figure 4I). In this approach, PRMT5 enhanced PMS2 transcription by binding to the ChIP‐2 promoter region (between −538 and −283 bp) upon CPT‐11 treatment. Notably, PRMT5 deletion markedly decreased the occupancy of the PMS2 promoter (Figure 4J). Consistently, H3R2me2s were enriched in the ChIP‐2 promoter in a PRMT5‐dependent manner (Figure 4K). The analysis of ChIP‐2 promoter regions and PRMT5 uncovered a specific complementary sequence. PRMT5 significantly increased PMS2 promoter luciferase activity post‐CPT‐11 treatment, which was abolished upon mutation of the binding site (Figure S5F–H, Supporting Information). Furthermore, The absence of PMS2 was required to maintain a relatively low IC50 and high γ‐H2AX levels of CPT‐11 following PRMT5 knockdown in CRC cells (Figure 4L,M; Figure S5I–L, Supporting Information).

PMS2 has been shown to play a role in DSBs, and its deficiency is associated with human dMMR in CRC.^[^ ^31^ ^]^ PMS2 expression was nearly absent in mice treated with a combination of PRMT5i and CPT‐11, suggesting that this treatment may induce a state resembling that of dMMR in CRC (Figure 4N). Tumors from mice treated with either dimethyl sulfoxide (DMSO) or the dual‐drug combination were sorted for RNA‐seq analysis. First, mouse genes were converted to *Homo sapien* homologs to estimate stromal and immune cells in MAlignant Tumor tissues using expression data (ESTIMATE).^[^ ^32^ ^]^ The dual‐drug combination group exhibited higher ESTIMATE scores and increased immune cell abundance compared to the DMSO group (Figure 4O; Figure S5M,N, Supporting Information). Using the murine Microenvironment Cell Population counter (mMCP‐counter),^[^ ^33^ ^]^ an immune deconvolution algorithm designed for bulk RNA‐seq data, we observed a significant increase in CD3^+^ and CD8^+^ T cells in the combination treatment group, indicative of enhanced anti‐tumor immunity (Figure 4P; Figure S5O, Supporting Information). Subsequently, we employed Differential Expression and Pathway Analysis of Tumor Heterogeneity 2 (DEPTH2)^[^ ^34^ ^]^ to quantify intratumoral heterogeneity and evaluate immune‐related gene set scores in mouse tissues (GO: 00 50852, GO: 00 19882). The analysis showed that PRMT5i combined with CPT‐11 resulted in significantly higher diversity scores, T cell receptor signaling, and antigen presentation pathway scores compared to DMSO treatment, consistent with the highly mutational phenotype typical of dMMR tumors (Figure 4Q; Figure S5P,Q, Supporting Information).^[^ ^35^ ^]^ This suggests that combination therapy increases the likelihood of generating new tumor antigens that can be recognized by the immune system. RNA‐seq analysis revealed a decrease in MMR gene expression following the dual‐drug treatment (Figure 4R). Finally, to infer the MSI status of dual‐drug‐treated mouse tissues, PreMSIm analysis,^[^ ^36^ ^]^ a tool for predicting microsatellite instability (MSI) from mRNA data, was performed. As expected, compared with the DMSO treatment, the dual‐drug‐treated tissues were classified as MSI‐high (Figure 4S). Western blot analysis revealed that PRMT5 levels in human pMMR CRC tissues were significantly higher than those in PMS2‐deficient CRC tissues (Figure S5R, Supporting Information). Correlation analysis demonstrated a positive association between PRMT5 and PMS2 expression in MSS CRC patients (Figure S5S, Supporting Information). Collectively, these findings suggest that the dual‐drug combination strategy induces a state resembling dMMR in MSS CRC.

### 2.5 PMS2 Deficiency Activates the cGAS‐STING Signaling Pathway

**Figure 5:**
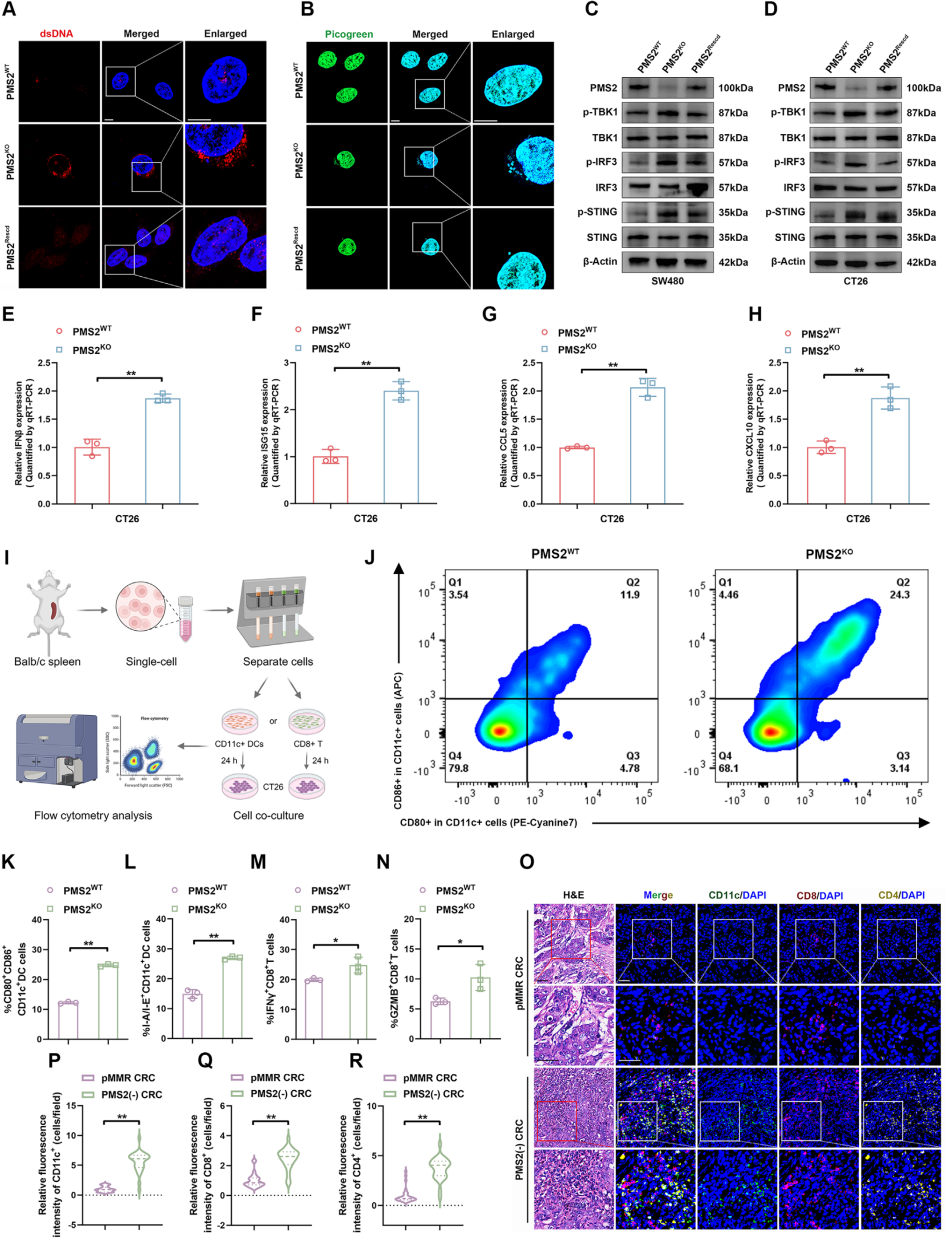
PMS2 deficiency promote cGAS/STING pathway activation in MSS CRC. A,B) Representative fluorescence images showing the cellular location and expression of dsDNA (A) and Picogreen (B) in PMS2^WT^, PMS2^KO^, and PMS2^rescued^ CRC cells. Scale bar = 5 µm. C,D) Western blot analysis of p‐TBK1, TBK1, p‐IRF3, IRF3, p‐STING, and STING levels in PMS2^WT^, PMS2^KO^ and PMS2^rescued^ SW480 (C) and CT26 (D) cells. E‐H) qRT‐PCR analysis showing the effect of PMS2 knockout on the expression of IFNβ (E), ISG15 (F), CCL5 (G), and CXCL10 (H) in CT26 cells. The *p*‐values were calculated using a two‐tailed Student's *t*‐test. I) Schematic diagram illustrating *Ex vivo* isolation of CD11c^+^ DCs and CD8^+^ T cells. The schematic illustration was created using Biorender. J) Representative flow cytometry images showing CD80^+^ and CD86^+^ expression on CD11c^+^ DCs in the PMS2^WT^ and PMS2^KO^ groups (*n* = 3). K,L) Expression levels of CD80 and CD86 (K), and I‐A/I‐E (L) on CD11c^+^ DCs in the PMS2^WT^ and PMS2^KO^ groups (*n* = 3). The *p*‐values were calculated using a two‐tailed Student's *t*‐test. M,N) Expression levels of IFNγ (M) and GZMB (N) on CD8^+^ T cells in the PMS2^WT^ and PMS2^KO^ groups (*n* = 3). The *p*‐values were calculated using a two‐tailed Student's *t*‐test. O–R) Representative fluorescence images (O) and quantitative analysis (P–R) of CD11c, CD8, and CD4 in pMMR and PMS2‐deficient CRC patients (*n* = 27). Fluorescence intensity normalized to control. The *p*‐values were calculated using a two‐tailed Student's *t*‐test. Scale bar = 50 µm. Error bars show the mean ± SD. ns, *p* > 0.05, *p < 0.05, **p < 0.01.

Although dMMR‐mediated immunotherapy responsiveness occurs via the activation of innate immune signaling, that is, the cGAS‐STING pathway^[^ ^16^ ^]^ it remains unclear whether PMS2 deficiency plays an essential role in the activation of this pathway. In order to analyze the immune activation profile, PMS2 wild‐type (PMS2^WT^), PMS2 knockout (PMS2^KO^), and PMS2 overexpression in PMS2^KO^ cells (PMS2^Rescd^) were generated in both human SW480 and mouse CT26 tumor cells, both of which are considered as MSS CRC cell lines. Anti‐dsDNA and PicoGreen staining were used to analyze DNA breaks and cytoplasmic DNA accumulation, both of which are essential for cGAS activation.^[^ ^37^ ^]^ The percentage of cells with cytosolic dsDNA accumulation was significantly higher in PMS2^KO^ cells than in PMS2^WT^ cells, and this accumulation was markedly reduced in PMS2^Rescd^ cells (**Figure** **5**A,B; Figure S6A,B, Supporting Information). These results suggested that PMS2 deficiency caused dsDNA to be released from the nucleus into the cytoplasm. Further analysis into whether PMS2^KO^‐mediated cytosolic dsDNA accumulation led to activation of the cGAS‐STING signaling pathway revealed that PMS2^KO^ cells exhibited increased phosphorylation of TANK‐binding kinase 1 (TBK1), interferon regulatory factor 3 (IRF3), and STING. The elevated phosphorylation levels were reversed in PMS2^Rescd^ cells (Figure 5C,D). Activation of the cGAS‐STING axis induces the expression of innate immune genes, including Interferon beta (IFNβ), interferon‐stimulated genes (ISGs), C–C motif chemokine ligand 5 (CCL5), and the C–X–C motif chemokine ligand 10 (CXCL10).^[^ ^16^, ^38^ ^]^ Significantly higher levels of IFNβ, ISG15, CCL5, and CXCL10 were observed in PMS2^KO^ cells compared to PMS2^WT^ cells in both SW480 and CT26 cell lines, suggesting that PMS2 deficiency positively regulates the activation of the cGAS‐STING signaling pathway in MSS CRC (Figure 5E–H; Figure S6C–F, Supporting Information).

Since IFNβ is a potent pro‐inflammatory mediator that recruits and activates dendritic cells (DCs),^[^ ^39^ ^]^ we isolated CD11c^+^ DCs from the spleens of Balb/c mice using anti‐mouse CD11c‐coated magnetic beads (Figure 5I). The isolated CD11c^+^ DCs were co‐cultured with PMS2^WT^ and PMS2^KO^ CT26 cells, respectively, to evaluate the potential adjuvant effects of PMS2 deficiency in CRC. After 24 h, markedly higher expression of mature DCs markers, including CD80, CD86, and I‐A/I‐E, within the CD11c^+^ population was observed in the PMS2^KO^ group (Figure 5J–L). DCs initiate the adaptive T cell immune response and are essential components of the immune system.^[^ ^40^ ^]^ Therefore, CD8^+^ T cells were isolated from mouse spleens and co‐incubated with PMS2^WT^ and PMS2^KO^ CT26 cells for 24 h (Figure 5I). Interestingly, greater activation of CD8^+^ T cell was observed in the PMS2^KO^ group, as indicated by higher levels of Interferon‐gamma (IFNγ) and Granzyme B (GZMB) expression compared to the PMS2^WT^ group (Figure 5M,N; Figure S6G,H, Supporting Information). Similarly, multiplexed immunofluorescence (mIF) analysis confirmed that PMS2 deficiency, compared to pMMR status, was associated with increased infiltration of CD11c^+^ DCs, CD4^+^ T cells, and CD8^+^ T cells in patients with CRC (Figure 5O–R). Collectively, these data suggested that PMS2 deficiency increased the leakage of nuclear DNA into the cytoplasm, thereby enhancing anti‐tumor immune responses.

### 2.6 PRMT5 Inhibition Combined with CPT‐11 Promotes Activation of Innate Immune Signaling in a PMS2‐Dependent Manner

**Figure 6:**
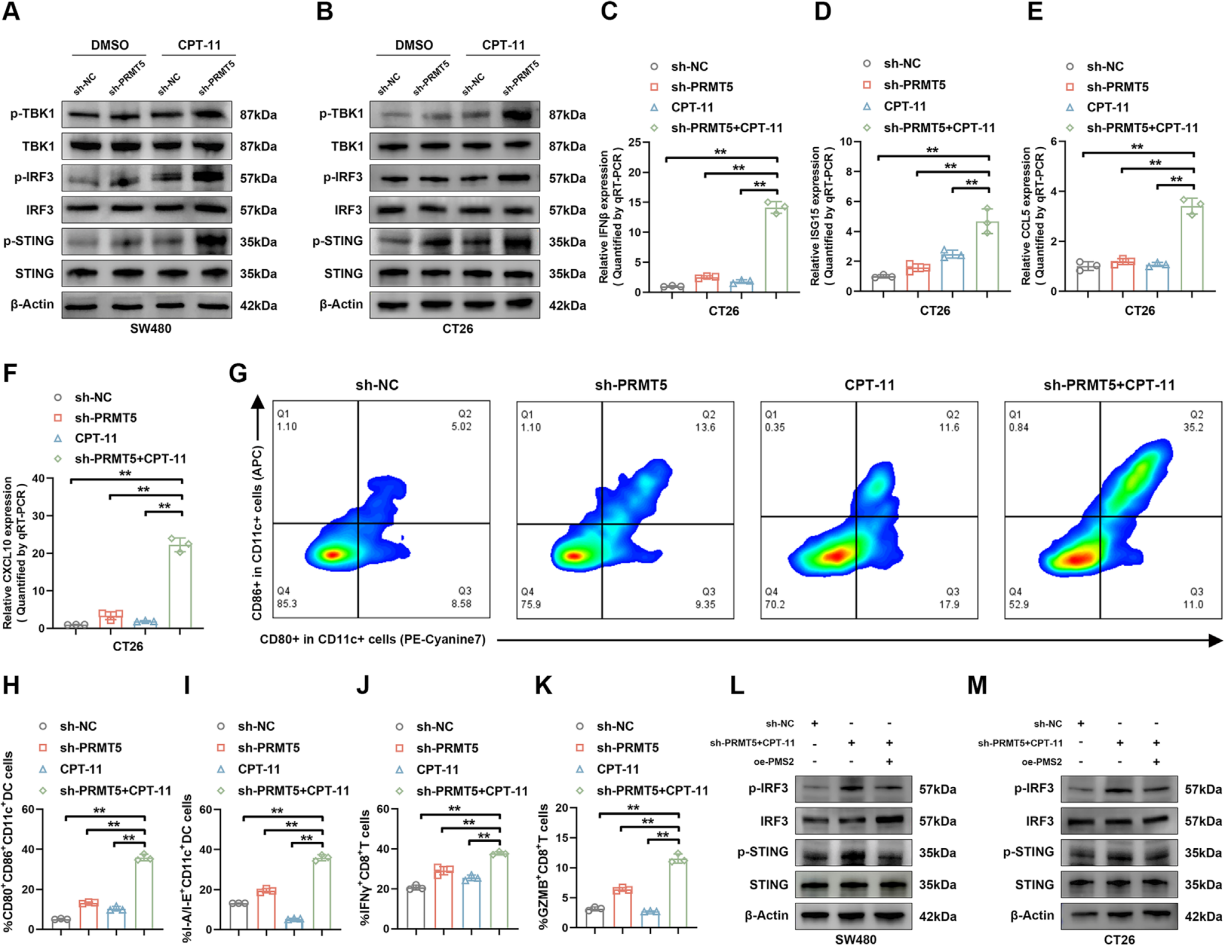
Combinational of PRMT5 silencing and CPT‐11 activates cGAS/STING pathway via restrain PMS2 expression. A,B) Western blot analysis showing the levels of p‐TBK1, TBK1, p‐IRF3, IRF3, p‐STING, and STING in SW480 (A) and CT26 (B) cells treated with control, PRMT5 silencing, CPT‐11, and PRMT5 silencing combined with CPT‐11. C–F) qRT‐PCR analysis of IFN‐β (C), ISG15 (D), CCL5 (E), and CXCL10 (F) levels in CT26 cells across all groups. The *p*‐values were calculated using one‐way ANOVA. G) Representative flow cytometry images showing CD80^+^ and CD86^+^ expression on CD11c^+^ DCs in the indicated groups (*n* = 3). H,I) Expression levels of CD80 and CD86 (H), and I‐A/I‐E (I) on CD11c^+^ DCs across all groups (*n* = 3). The *p‐*values were calculated using one‐way ANOVA. J,K) Expression levels of IFNγ (J) and GZMB (K) on CD8^+^ T cells in the indicated groups (*n* = 3). The *p*‐values were calculated using one‐way ANOVA. L,M) Western blot analysis showing the effect of PRMT5 silencing combined with CPT‐11 on PMS2 overexpression‐induced cGAS‐STING pathway inactivation in SW480 (L) and CT26 (M) cells. Error bars show the mean ± SD. ns, *p* > 0.05, *p < 0.05, **p < 0.01.

Next, we investigated whether PRMT5 inhibition combined with CPT‐11 positively regulated the activation of the cGAS‐STING signaling pathway. Notably, enhanced phosphorylation levels of TBK1, IRF‐3, and STING, along with increased expression of innate immune genes, were observed in both PRMT5‐inhibited and CPT‐11‐treated SW480 and CT26 cells, but not in the control groups (**Figure** **6**A–F; Figure S7A–D, Supporting Information). Flow cytometry was performed to investigate the antigen‐presenting capability of CD11c^+^ DCs across all groups. The results showed that compared to the control group, the expression levels of CD80, CD86, and I‐A/I‐E on CD11c^+^ DCs from mouse spleens were significantly upregulated when co‐cultured with CT26 cells in the CPT‐11‐treated PRMT5 inhibition group (Figure 6G–I). There was also a marked increase in the activation of CD8^+^ T cells in mouse spleens when incubated with PRMT5‐silenced CT26 cells treated with CPT‐11. Specifically, 38.7% of CD8^+^ T cells expressed IFNγ, and 12.4% expressed GZMB; in the absence of PRMT5 inhibition and CPT‐11 treatment, only 19.8% of CD8^+^ T cells expressed IFNγ, and 3.2% expressed GZMB (Figure 6J,K; Figure S7E,F, Supporting Information). Through CellChat analysis of scRNA‐seq data from MSS CRC patients, we found that the group with the highest PRMT5 expression exhibited the lowest interaction levels between DCs and T cells (Figure S7G, Supporting Information). Furthermore, PMS2 overexpression successfully reversed the PRMT5 inhibition with CPT‐11 treatment‐induced upregulation of IRF3 and STING phosphorylation, indicating that the combined treatment activated the cGAS‐STING signaling pathway by downregulating PMS2 (Figure 6L,M).

### 2.7 The Dual‐Drug Combination Therapy Enhances Anti‐Tumor Immunity while Upregulating TIGIT Expression in MSS CRC

**Figure 7:**
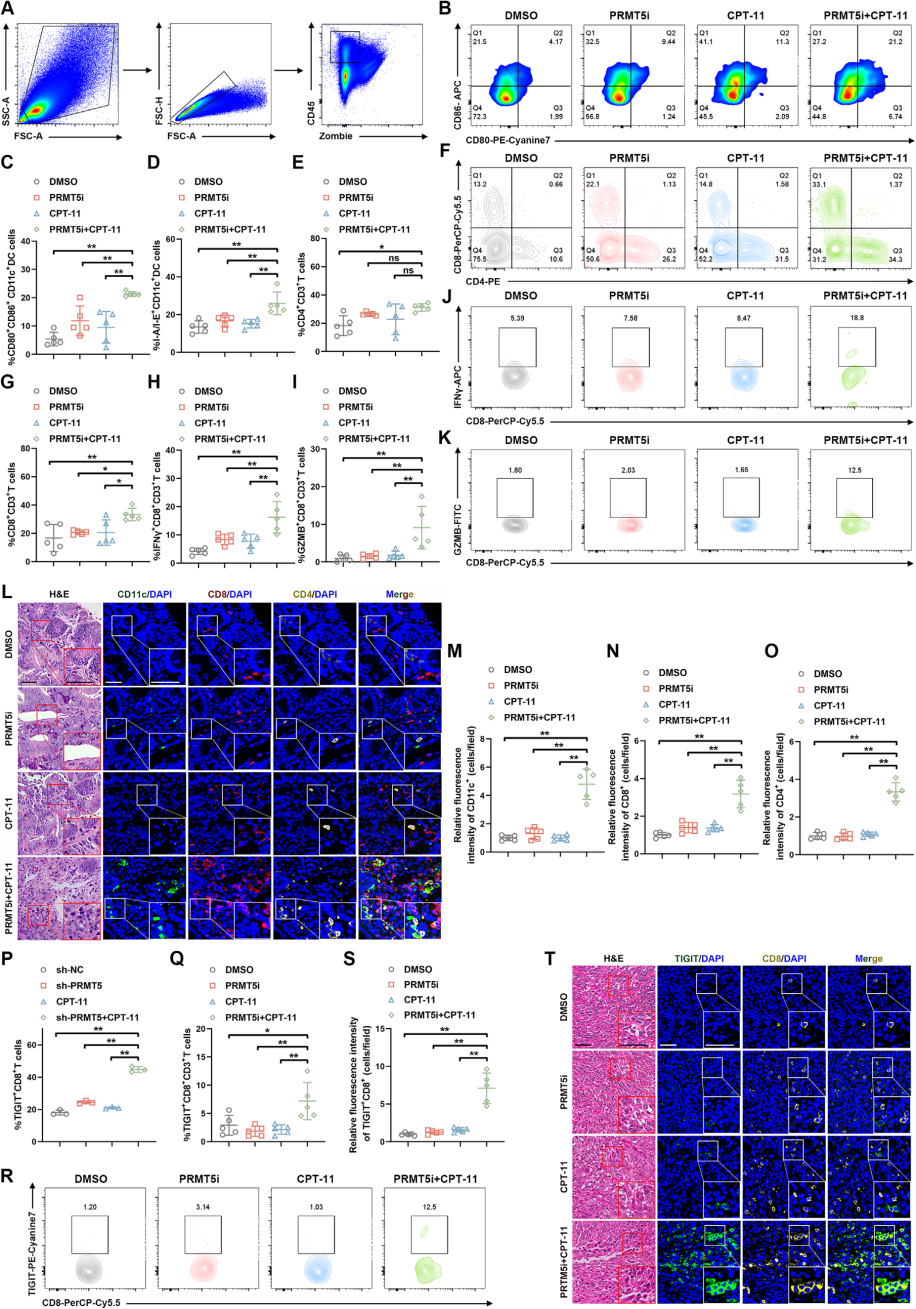
The dual‐drug combination therapy elevates the level of TIGIT in MSS CRC. A) Workflow illustrating the isolation of CD45^+^ live cells from a single‐cell suspension for further analysis. B–D) Representative flow cytometry images of CD80^+^ and CD86^+^ expression (B), and expression levels of CD80 and CD86 (C), and I‐A/I‐E (D) on CD11c^+^ DCs isolated from tumor‐bearing mice treated with DMSO, PRMT5i, CPT‐11, or PRMT5i + CPT‐11 (*n* = 5). The *p*‐values were calculated using one‐way ANOVA. E–G) Representative flow cytometry images (F) and quantitative analysis (E,G) of CD4^+^ and CD8^+^ on CD3^+^ T cells in the indicated mice (*n* = 5). The *p*‐values were calculated using one‐way ANOVA. H–K) Representative flow cytometry images (J,K) and quantitative analysis (H,I) of IFNγ^+^ and GZMB^+^ on CD8^+^ CD3^+^ T cells in the indicated tumor tissues (*n* = 5). The *p‐*values were calculated using one‐way ANOVA. L–O) Representative fluorescence images (L) and quantitative analysis (M–O) of CD11c, CD8, and CD4 in the indicated mouse tissues. Fluorescence intensity normalized to control. The *p*‐values were calculated using one‐way ANOVA. Scale bar = 50 µm. P) levels of TIGIT on CD8^+^ T cells isolated from the spleens of mice in the indicated groups (*n* = 3). The *p*‐values were calculated using one‐way ANOVA. Q,R) Representative flow cytometry images (R) and quantitative analysis (Q) of TIGIT on CD8^+^ T cells isolated from the tumor tissues of the indicated mice (*n* = 5). The *p*‐values were calculated using one‐way ANOVA. S,T) Representative fluorescence images (T) and quantitative analysis (S) of TIGIT levels on CD8^+^ cells in the indicated mouse tissues. Fluorescence intensity normalized to control. The *p*‐values were calculated using one‐way ANOVA. Scale bar = 50 µm. Error bars show the mean ± SD. ns, *p *> 0.05, *p < 0.05, **p < 0.01.

The role of dual‐drug combination therapy with PRMT5i and CPT‐11 in the activation of anti‐tumor immunity was further investigated in vivo. Consistent with in vitro results, dual‐drug therapy stimulated the expression of CD80, CD86, and I‐A/I‐E in CD11c^+^ DCs of CT26 tumors (**Figure** **7**A–D). The levels of CD4^+^ and CD8^+^ tumor‐infiltrating lymphocytes, as well as CD8^+^ T cell effector molecules IFNγ and GZMB, within the TME also dramatically increased in tumor‐bearing mice with dual‐drug therapy treatment (Figure 7E–K). Meanwhile, tumor‐infiltrating CD11c^+^ DCs, CD4^+^ T cells, and CD8^+^ T cells were also upregulated following PRMT5i treatment combined with CPT‐11 (Figure 7L–O). Furthermore, CD8^+^ T cell‐specific depletion in CT26 tumor‐bearing mice markedly reduced the anti‐tumor efficacy of the dual‐drug combination therapy, suggesting a mechanistic dependence on CD8^+^ T cell‐mediated immune activation (Figure S8A–D, Supporting Information).

The cGAS/STING signaling‐induced activation of the innate immune system may synergize with ICIs to amplify anti‐tumor efficacy.^[^ ^41^ ^]^ Therefore, subsequent experiments evaluated whether the dMMR‐like state mediated by the dual‐drug combination therapy could sensitize tumors to αPD‐1 therapy. Surprisingly, αPD‐1 treatment did not confer any reductions in tumor volume nor weight (Figure S8E–H, Supporting Information). Previous studies have demonstrated that CD8^+^ T cells within CT26 mouse tumors exhibit a PD‐1^+^ exhausted phenotype.^[^ ^42^ ^]^ mIF confirmed that the combination therapy did not induce PD‐1 upregulation (Figure S8I,J, Supporting Information). Flow cytometry analysis revealed an increased expression of TIGIT and TIM3 in CD8^+^ T cells following the combined intervention in ex vivo experiments, whereas LAG3 expression remained unchanged (Figure 7P; Figure S8K–M, Supporting Information). Among these immunosuppression receptors, the upregulation of TIGIT in CD8^+^ T cells was particularly significant (Figure 7Q–T). Furthermore, The combination treatment significantly upregulated CD155, a ligand of TIGIT, in mouse tumors without altering CD112 levels, suggesting a positive correlation between TIGIT expression and CD155 in the TME (Figure S8N,O, Supporting Information). Previous studies have demonstrated that prolonged stimulation induces persistent T cell activation, ultimately leading to T cell exhaustion, whereas chronic antigen stimulation transiently maintains the survival and memory phenotype of stem‐like T cells via activation of the cGAS‐STING pathway.^[^ ^43^, ^44^ ^]^ To investigate whether the dual‐drug combination‐induced upregulation of TIGIT on CD8^+^ T cells leads to functional impairment, mIF analysis revealed a significant increase in the proportion of TCF1^+^ CD8^+^ T cells, known as stem‐like T cells, indicating that the T cell population retains the potential to differentiate into effector T cells despite an elevated proportion of exhausted T cells (Figure S8P–R, Supporting Information). These findings suggest that the dMMR‐like state induced by dual‐drug treatment promotes a mixed‐exhaustion state of T cells under chronic antigen exposure in MSS CRC.

### 2.8 The dMMR‐Like State in MSS CRC Is Sensitive to Anti‐TIGIT Therapy

**Figure 8:**
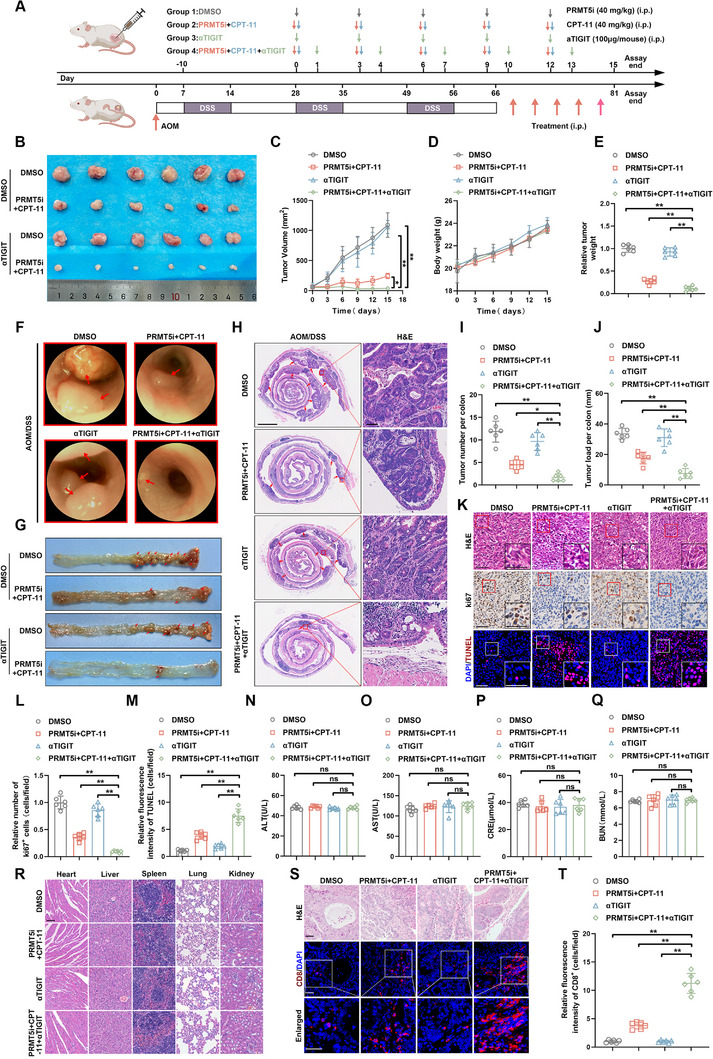
The dual‐drug combination therapy induced a dMMR‐like response to anti‐TIGIT therapy. A) Timeline schematic showing the treatment of subcutaneous syngeneic tumor mice and AOM/DSS‐induced tumor‐bearing mice. B–E) Representative tumor images (B), tumor growth curves (C), body weight (D), and relative tumor weight (E) of subcutaneous syngeneic tumor mice treated with DMSO, PRMT5i (40 mg kg^−1^) + CPT‐11 (40 mg kg^−1^), αTIGIT (100 µg per mouse), or PRMT5i + CPT‐11 + αTIGIT (*n* =  6). The *p*‐values were calculated using two‐way ANOVA (C) and one‐way ANOVA (E). F–J) Representative mini‐endoscopy images (F), tumor images (G), H&E staining (H), tumor numbers (I), and tumor load (J) of AOM/DSS‐induced tumor‐bearing mice treated with the indicated therapies (*n* = 6). Scale bar (left part) = 2 mm, Scale bar (right part) = 50 µm. The *p*‐values were calculated using one‐way ANOVA. K–M) Representative images (K) and quantitative analysis (L,M) of ki‐67 and TUNEL staining in tumor tissues from the indicated groups. The *p*‐values were calculated using one‐way ANOVA. Scale bar = 50 µm. N–Q) Levels of ALT (N), AST (O), CRE (P), and BUN (Q) in blood from AOM/DSS‐induced mice in the indicated groups. The *p‐*values were calculated using one‐way ANOVA. R) Representative images of organ indexes, including heart, liver, spleen, lungs, and kidneys, in AOM/DSS‐induced mice. Scale bar = 50 µm. S,T) Representative fluorescence images (S) and quantitative analysis (T) of CD8^+^ cells in the indicated mouse tissues. The *p*‐values were calculated using one‐way ANOVA. Scale bar = 50 µm. Error bars show the mean ± SD. ns, *p* > 0.05, *p < 0.05, **p < 0.01.

**Figure 9:**
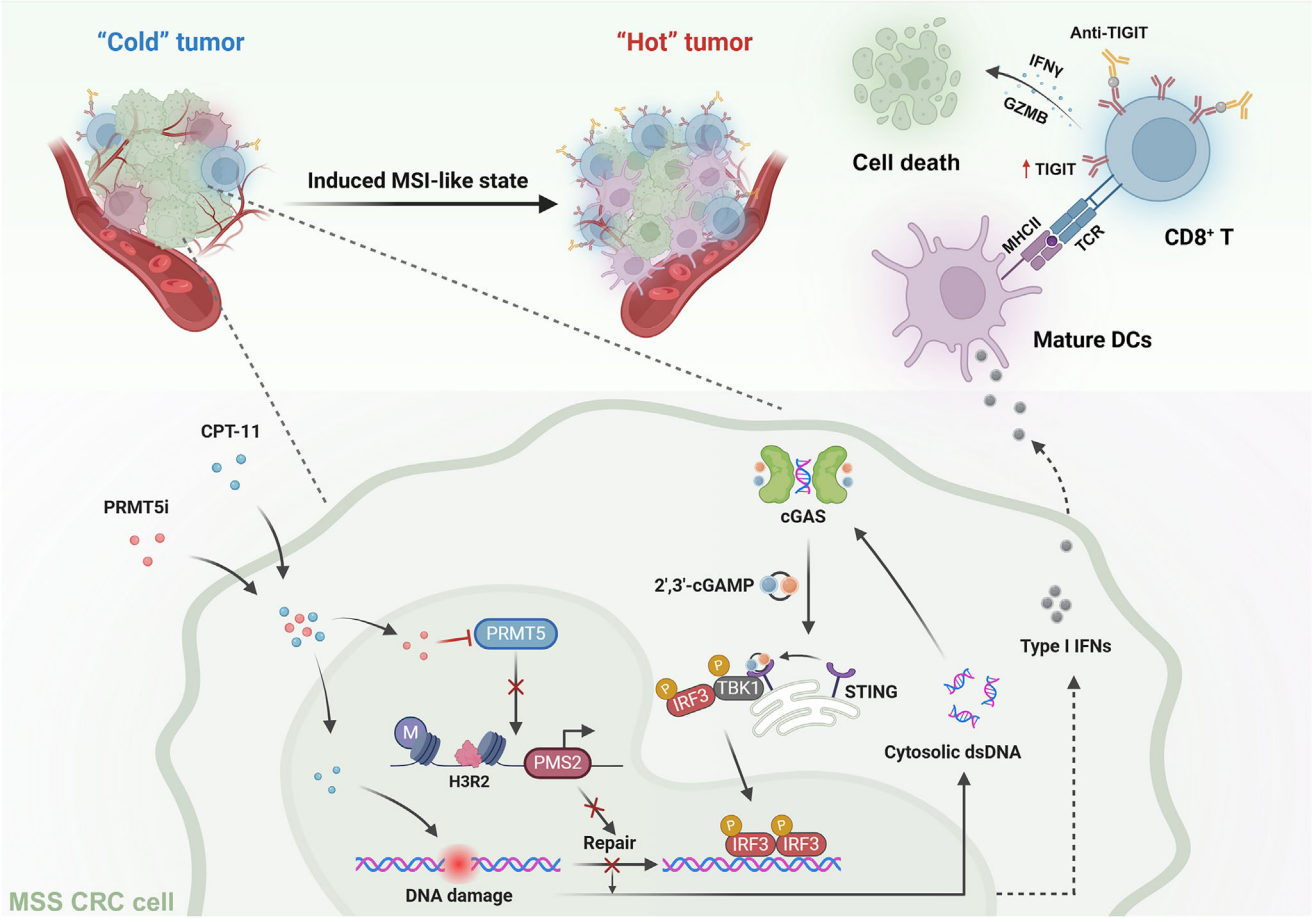
Schematic illustrating the potential mechanism by which the combination of PRMT5i and CPT‐11 elicited a dMMR‐like state and enhanced αTIGIT therapy in MSS CRC. The schematic illustration was created using Biorender.

Given that mixed‐exhausted T cells retain partial functionality and may restore their effector potential through immunotherapy,^[^ ^45^ ^]^ we investigated whether the combination of PRMT5i, CPT‐11, and αTIGIT would have synergistic anti‐tumor effects in the MSS mouse model. To this aim, Balb/c mice bearing CT26 tumors and AOM/DSS‐induced CRC were treated with DMSO, PRMT5i combined with CPT‐11, αTIGIT, or the combination of all three (**Figure** **8**A). While PRMT5i combined with CPT‐11 partially reduced tumor volume and burden compared to DMSO or αTIGIT alone, the combination of PRMT5i, CPT‐11, and αTIGIT achieved the most significant reduction in tumor volume and burden in both the subcutaneous and AOM/DSS mouse models (Figure 8B–J). Survival analysis in the subcutaneous tumor model demonstrated that the three‐drug combination therapy significantly prolonged mouse survival (Figure S8S, Supporting Information). In addition, the three‐drug combination group exhibited the lowest levels of ki67 and the highest levels of TUNEL staining, indicating effective inhibition of cell proliferation and increased apoptosis (Figure 8K–M). This treatment did not alter blood biochemical markers, including alanine aminotransferase (ALT), aspartate aminotransferase (AST), blood creatinine (CRE), and blood urea nitrogen (BUN), nor did it affect organ indices for the heart, liver, spleen, lungs, and kidneys, suggesting the safety of the three‐drug combination in mice (Figure 8N–R). Ultimately, this synergistic effect was associated with increased infiltration of CD8^+^ T cells into tumor tissues, indicating the successful reactivation of T cell immunity (Figure 8S,T). Taken together, these findings support the notion that combination treatment with PRMT5i, CPT‐11, and TIGIT facilitates tumor growth inhibition and represents a novel intervention strategy for MSS CRC (**Figure** **9**).

## 3 Discussion

Epigenetic‐based therapies target transcriptional programming, modulating diverse signaling pathways in cancer cells and ultimately determining the fate of these cell populations.^[^ ^46^ ^]^ The role of epigenetic therapy in chemotherapy‐mediated DNA damage has attracted increasing attention. By enhancing their DDR capacity, tumor cells can evade cell death, resulting in heightened resistance to chemotherapy and radiotherapy.^[^ ^47^, ^48^ ^]^ However, the mechanisms by which targeting epigenetic factors influences chemotherapy‐mediated DNA damage in MSS CRC remain poorly understood. Here, we provide evidence that PRMT5 is a significant prognostic factor for MSS CRC progression and is an important DDR‐related epigenetic marker based on scRNA‐seq analysis of MSS CRC patients and bulk RNA‐seq analysis of paired MSS CRC tissues and NATs. Previous studies have suggested that epigenetic modifications enhance chemotherapy‐induced tumor antigen exposure;^[^ ^49^ ^]^ however, the mechanisms underlying epigenetic‐mediated immune activation in MSS CRC remain unclear. In the present study, a novel mechanism was revealed whereby PRMT5 inhibition promotes CPT‐11 sensitivity and synergistically induces a dMMR‐like state in MSS CRC, further activating the cGAS‐STING signaling pathway and boosting anti‐tumor immunity. We highlight the unique contributions of our study from the perspective of epigenetic therapy in MSS CRC combination strategies.

MSS and MSI represent distinct molecular subtypes of CRC, and the dynamic nature of tumor evolution and the plasticity of the TME suggests that these categories are not always rigidly defined.^[^ ^50^ ^]^ Studies have shown that MSS tumors do not react uniformly, with some demonstrating responsiveness to ICB therapy.^[^ ^8^ ^]^ Understanding how MSS CRC can exhibit MSI‐like features is crucial for transforming immunologically “cold” tumors into “hot” tumors.^[^ ^51^ ^]^ Alternative pathways independent of MMR deficiency may also drive genomic instability. Recent research suggests that certain MSS tumors may acquire MSI‐like features through EGFR/BRAF inhibition.^[^ ^52^ ^]^ In this study, we propose a novel combination therapy using PRMT5i and CPT‐11, which enhances anti‐tumor immunity by leveraging epigenetic modifications that mediate PMS2 deficiency in MSS CRC. Further research is needed to identify biomarkers that can predict which cases of MSS CRC are likely to develop MSI‐like characteristics.

MMR‐associated genes can be regulated by epigenetic‐based therapies.^[^ ^46^ ^]^ This may lead to a scenario in which, despite the genetic machinery remaining intact, the MMR gene becomes functionally inactive, thereby mimicking the effects observed in MSI tumors. Approved epigenetic inhibitors are limited to hematological cancers, and the weak response of solid tumors to monotherapy is one of the factors that restrict the use of epigenetic anti‐tumor drugs.^[^ ^53^ ^]^ Despite this, there has been limited research on potential mechanisms to overcome the limitations of monotherapy with epigenetic inhibitors in MSS CRC. This study showed that GSK‐3326595, a phase II clinical drug targeting PRMT5, synergistically inhibited tumor growth when combined with chemotherapy in both subcutaneous tumor and AOM/DSS models in Balb/c mice. To ensure safety, injections were administered every three days to allow for hematopoietic recovery between treatments, despite the main problem of PRMT5 inhibitors being hematological toxicity.^[^ ^54^ ^]^ Reports suggest that combination therapy can modify intrinsic or extrinsic tumor factors, thereby enhancing the success rate of anticancer immunotherapy.^[^ ^53^, ^55^ ^]^ Similarly, our study confirmed that PRMT5 promotes DNA damage repair through epigenetic modifications. In contrast, the inhibition of PRMT5 mediated the release of more dsDNA into the cytoplasm following CPT‐11 treatment in a PMS2‐dependent manner. This released dsDNA binds to cGAS, promoting IFNβ secretion and further enhancing anti‐tumor T cell immunotherapy through DCs‐T‐cell‐dependent functions. Collectively, these results overcome the limitations of monotherapy with epigenetic inhibitors and expand knowledge regarding the role of combination therapy in enhancing anti‐tumor immunity in MSS CRC.

Recent studies have demonstrated that prolonged stimulation by chemotherapeutic agents induces persistent T cell activation, ultimately driving T cells into an exhausted state.^[^ ^43^ ^]^ In the early stages of exhaustion, T cells may adopt a mixed‐exhaustion phenotype, characterized by retained potential to differentiate into effector T cells but diminished responsiveness to antigenic stimulation.^[^ ^55^ ^]^ In the current study, PRMT5i combined with CPT‐11 activated DCs, thereby enhancing CD8^+^ T cell activation. The combination treatment induced dMMR‐like characteristics in Balb/c mice and significantly upregulated TIGIT expression on CD8+ T cells, while simultaneously increasing the proportion of stem‐like T cells. These findings suggest that the combination therapy may induce a partial mixed‐exhaustion state in T cells, while simultaneously creating a potential opportunity for synergistic treatment with αTIGIT. TIGIT, a novel immune checkpoint, plays a role in immune suppression and facilitates tumor immune evasion by inhibiting immune cell functions.^[^ ^57^ ^]^ Notably, combining IL‐15 with TIGIT blockade can activate CD8^+^ TIL‐mediated anti‐tumor immunity in lung adenocarcinoma.^[^ ^58^ ^]^ However, the role of TIGIT in the development and progression of MSS CRC remains unclear. In this study, we demonstrated that the triple combination of a PRMT5 inhibitor, CPT‐11, and αTIGIT exhibited superior anti‐tumor efficacy with minimal toxic side effects, and this synergistic effect successfully reactivated T cell immunity. Therefore, this novel triple‐drug combination therapy strategy offers significant therapeutic potential for patients with MSS CRC resistant to immunotherapy. However, given the significant heterogeneity of MSS CRC, large‐scale clinical trials, complemented by biomarker profiling, are essential to confirm these findings and to identify the optimal patient subgroups for this treatment strategy.

## 4 Conclusion

In summary, our study demonstrated that PRMT5 blockade enhances CPT‐11 sensitivity in MSS CRC by inhibiting PMS2 expression. Furthermore, the combination of PRMT5i with CPT‐11 elicited a dMMR‐like state and activated cGAS‐STING signaling in a PMS2‐dependent manner, which subsequently enhanced CD8^+^ T cell‐mediated anti‐tumor immunity and increased sensitivity to αTIGIT treatment. Clinically, we propose the combined use of PRMT5i, CPT‐11, and αTIGIT as a potential therapeutic strategy for MSS CRC. To the best of our knowledge, this is the first report to reveal a dMMR‐like‐dependent mechanism regulated by a combination of epigenetic inhibitors and chemotherapeutic drugs to enhance sensitivity to immunotherapy, which may offer a novel intervention strategy to address immunotherapy resistance in MSS CRC.

## 5 Experimental Section

Additional materials and methods can be found in Supporting Information.

### Clinical Samples and Ethics Statement

All human tissue samples from patients with MSS CRC who underwent surgical resection were obtained at Xiangya Hospital of Central South University (Changsha, China) between 2019 and 2023. These samples were used for scRNA‐seq, bulk RNA‐seq, and IHC. Each patient's diagnosis was independently confirmed by three pathologists. The study was performed in accordance with the Helsinki Declaration and Rules of Good Clinical Practice. Informed consent was obtained from the recruited patients, and the collection of human tissue was approved by the Ethics Committee of Xiangya Hospital, Central South University [approval number: 201 905 131].

### Sequence Preparation of MSS CRC Patient Cohort

For scRNA‐seq, fresh tumor tissue samples from three MSS CRC patients were collected and dissociated into single cells using microwells with no fewer than 200 000 cells of a specific size. Single‐cell suspensions were then loaded onto the BD Rhapsody single‐cell analysis system for cell capture, and RNA was reverse‐transcribed to generate cDNA libraries. The prepared libraries were subjected to PE150 sequencing on the Illumina NovaSeq ^6000^ platform.

For bulk RNA‐seq, total RNA was extracted from 49 fresh MSS CRC tissue samples using the TRIzol reagent (Invitrogen, Carlsbad, CA, USA). Subsequently, the first and second strands of the cDNA were synthesized for PCR amplification and library construction. The library was sequenced using the Illumina NovaSeq ^6000^ platform.

### Cell Lines and Cell Culture

CRC cell lines (SW480, SW620, DLD1, LS513, LOVO, RKO, CACO‐2, and CT26), normal human colon mucosal epithelial cells (NCM460), and human embryonic kidney cells (HEK‐293T) were acquired from the Institute of Biomedical Sciences (IBS, Shanghai, China). SW620, DLD1, LS513, RKO, LOVO, CT26, and NCM460 cells were cultured in 1640 medium (Corning). SW480 cells were maintained in McCoy's 5A medium (Procell, Wuhan, China), CACO‐2 cells were cultured in minimum essential medium (MEM) (Procell, Wuhan, China), and HEK‐293T cells in Dulbecco's modified Eagle medium (DMEM) (Corning, Shanghai, China). All media were supplemented with 10% fetal bovine serum (FBS) (Cellmax, Lanzhou, China) and maintained at 37 °C in a humidified incubator with 5% CO_2_.

### Chromatin Immunoprecipitation (ChIP)

ChIP assays were conducted with the EZ‐Magna ChIP A/G kit (Merck Millipore, Billerica, MA, USA). Briefly, CRC cells were stimulated with CPT‐11 (50 µm) for 24 h. A total of 1 × 10⁷ cells were collected and crosslinked at room temperature with 4% paraformaldehyde for 10 min, followed by chromatin separation using cell lysis buffer and nuclear lysis buffer. Subsequently, chromatin was sonicated into 500–800 bp fragments and incubated with antibodies against control IgG, PRMT5, and H3R2me2s. The antibody‐bound chromatin complexes were immunoprecipitated overnight at 4 °C using Protein A/G‐coated magnetic beads. DNA was then eluted, purified from the beads, and analyzed by qRT‐PCR. Detailed information on the antibodies used in the experiments is provided in Table S3 (Supporting Information), and the primer sequences used for the ChIP qRT‐PCR are listed in Table S1 (Supporting Information).

### Experimental Animal Models and Treatments

All animal studies were carried out in accordance with the Medical Experimental Animal Care Commission of Central South University.

For the subcutaneous tumor model, male Balb/c mice aged 4–6 weeks were obtained from the Experimental Animal Center (Changsha, China). The mice were randomly divided into groups and 5 × 10⁶ CT26 cells were subcutaneously inoculated into the dorsal side of each mouse. When the tumors reached ≈200 mm^3^, mice were treated with DMSO (1%, Solarbio, Beijing, China), PRMT5i (40 mg kg^−1^, Selleck Chemicals, Houston, TX, USA), CPT‐11 (40 mg kg^−1^, Medchemexpress, Shanghai, China), 5‐FU (40 mg kg^−1^, Medchemexpress, Shanghai, China), Oxaliplatin (2.5 mg kg^−1^, Medchemexpress, Shanghai, China), αPD‐1 (100 µg mouse^−1^, Selleck Chemicals, Houston, TX, USA), αTIGIT (100 µg mouse^−1^, Selleck Chemicals, Houston, TX, USA), or αCD8 (200 µg mouse^−1^, Selleck Chemicals, Houston, TX, USA) by intraperitoneal injection every three days. The tumor size and tumor weight were measured during the treatment period. To assess the percent survival of animals, mice bearing tumors exceeding 2000 mm^3^ were defined as “dead”.

For the AOM/DSS model, male Balb/c mice aged 5–8 weeks were subcutaneously injected with an AOM solution (10 mg kg^−1^, Medchemexpress, Shanghai, China). Seven days later, 2.5% dextran sulfate sodium (DSS) (Medchemexpress, Shanghai, China) was administered in the drinking water for 7 days, followed by 2 weeks of regular drinking water. This DSS treatment cycle was repeated twice for a total of three cycles. After the three cycles, intraperitoneal injections of DMSO, PRMT5i, CPT‐11, or αTIGIT were administered every three days. The tumor number and diameter were monitored using mini‐endoscopy, and the tumor load was calculated as the cumulative tumor diameter in millimeters per mouse.

### Sorting of CRISPR/Cas9‐Induced Knockout Cells

CRISPR‐Cas9‐mediated plasmids expressing short guide RNA (sgRNA) targeting the PMS2 gene (Genechem, Shanghai, China) and negative control (Genechem) were synthesized and cloned into the GV464 (phU6‐sgRNA‐SpCas9‐2A‐EGFP) vector via BsmBI sites. These plasmids were subsequently transfected into SW480 and CT26 cells. Single tumor cells expressing GFP fluorescence were sorted using a BD FACS Aria II Flow Cytometer (BD Biosciences, San Jose, CA, USA) and subsequently aliquoted into 96‐well plates to construct PMS2 knockout CRC cell lines.

### Multiplexed Immunofluorescence (mIF)

Human or mice tumor tissue sections were deparaffinized and subjected to antigen retrieval. A series of primary antibodies targeting different cell markers were sequentially applied, followed by a species‐specific secondary antibody conjugated to a distinct fluorophore. The slides were subsequently scanned using an Akoya Vetra Polaris system (Akoya Biosciences).

### Statistical Analysis

All statistical analyses were performed using GraphPad Prism (version 9.0) and R software (version 4.3.2). Error bars in the graphs represent the standard deviation of three independent experiments. The significance between two groups was analyzed using a two‐tailed unpaired or paired Student's *t*‐test, whereas the variance among multiple groups was assessed using one‐way analysis of variance (ANOVA) and two‐way ANOVA. Spearman's correlation analysis was used to evaluate the relationship between the two molecules. Statistical significance was set at *p* < 0.05.

### Ethics Approval Statement

The collection of human tissues was approved by the Ethics Committee of Xiangya Hospital, Central South University [approval number: 201905131]. The study was performed in accordance with the Helsinki Declaration and Rules of Good Clinical Practice. Informed consent was obtained from all included patients.

All animal studies were carried out in accordance with the Medical Experimental Animal Care Commission of Central South University.

## Conflict of Interest

The authors declare no conflict of interest.

## Author Contributions

J.Z., Y.H., and S.Z. designed the study. J.Z., X.Z., and H.Z. conducted the in vitro and in vivo experiments. S.F. and G.C. performed data analyses. Y.P. and D.T. conducted clinical data analyses. J.Z., H.Z., and F.Z. performed IF and IHC experiments. J.Z., Y.H., and H.S. wrote the manuscript. All the authors have read and approved the final version of the manuscript.

## Supporting information



Supporting Information

Supporting Information

## Data Availability

The data that support the findings of this study are available from the corresponding author upon reasonable request.
