# Supplementary material for: PRMT5 Inhibitor Synergizes with Chemotherapy to Induce Resembling Mismatch Repair Deficiency and Enhance Anti‐TIGIT Therapy in Microsatellite‐Stable Colorectal Cancer
Source: Adv Sci (Weinh). 2025 May 8;12(27):2500271. doi: 10.1002/advs.202500271 (PMC12279233; doi:10.1002/advs.202500271)
Supplement: Supplementary file 1 — Supporting Information [file ADVS-12-2500271-s002.docx]

Supporting Information

**PRMT5 Inhibitor Synergizes with Chemotherapy to Induce Resembling Mismatch Repair Deficiency and Enhance Anti-TIGIT Therapy in Microsatellite-Stable Colorectal Cancer**

*Jiang Zhu, Shenao Fu, Xi Zou, Hanjiang Zeng, Guangzu Cui, Yinghui Peng, Diya Tang, Fan Zhang, Hong Shen*, Shan Zeng*, Ying Han**

**Supplementary** Experimental Section

*Epigenetic expression and DDR status analysis in the MSS CRC patient cohort*: For bulk RNA-seq, we used TPM values to calculate and compare the average expression of genes related to epigenetic regulation across the samples.

For single-cell RNA-seq, the R package Seurat (version 5.1.0) was employed for quality control and bioinformatic analyses. Cells with 200-5000 detected genes and less than 20% mitochondrial content were retained. Following data integration using the Harmony method, the top 15 principal components were utilized to construct a K-nearest neighbor (KNN) graph. We used the FindClusters functions to construct a shared nearest neighbor (SNN) graph and identify cell clusters. Clusters were identified with the resolution parameter set to 1. Nonlinear dimensionality reduction was performed using UMAP for visualization purposes. Cell types within each cluster were determined based on known marker gene expression, and only epithelial cells were selected for further analysis. After a second round of integration and dimensional reduction with same workflow, clusters were re-identified with the resolution parameter set to 0.5. The R package inferCNV (version 1.18.1) was used to assess copy number variations (CNVs) in each cell. The CNV profiles of tumor cells, along with their CNV scores, were combined to facilitate the identification of malignant cells. The DDR score of each cell was calculated using the R package AUCell (version 1.22.0) with DDR genes from a previous study. Spearman correlation analysis was performed to evaluate the relationship between DDR scores and gene expression. Genes with a P-value of less than 0.05 were considered DDR-related in the single-cell RNA-seq data.

Cell communication analysis was performed with R package CellChat (version 1.6.1).

*Plasmids transfection and Lentiviral transduction*: To construct stable knockdown or overexpression of PRMT5 and PMS2, respectively, in SW480, SW620, and CT26 cell lines, the short hairpin RNA (shRNA) (Genechem, Shanghai, China) or overexpression plasmids (Genechem, Shanghai, China) were co-transfected with PEI and packaging plasmids into HEK-293T cells to produce lentivirus. The culture supernatants of the transfected cells were collected at 48 and 72 h, then filtered through a 0.45 μm filter to remove cell debris. Polybrene (IGE, Guangzhou, China) was used to enhance infection efficiency, and the virus was subsequently used to infect CRC cells. Stable transfectants were selected using puromycin (Abiowell, Changsha, China). The transfection efficiency was assessed using real-time quantitative PCR (qRT-PCR). All sequences are provided in Table S2.

*Proteins extraction and Western blotting analysis*: Total proteins were extracted using RIPA lysis buffer (NCM Biotech, Suizhou, China) supplemented with 1% phosphatase and protease inhibitor cocktail (NCM Biotech, Suizhou, China) and quantified using the BCA Protein Assay Kit (Epizyme, Shanghai, China). Equal amounts of total proteins were separated by SDS-PAGE and transferred to polyvinylidene fluoride membranes (Merck Millipore, Billerica, MA, USA). Nonspecific binding sites were blocked with 5% bovine serum albumin (BSA) (Biosharp, Hefei, China). The membranes were incubated with the corresponding primary antibodies at 4 °C overnight, followed by incubation with horseradish peroxidase-conjugated secondary antibodies for 1 h the next day. Protein bands were visualized using the Amersham ImageQuant™ 800 (Cytiva, Japan) and quantified with ImageJ software (NIH, Bethesda, USA).

*Annexin V-FITC/PI apoptosis assay*: Apoptosis was detected using the Annexin V-FITC/PI Kit (4A Biotech, Beijing, China) according to the manufacturer’s protocol. Briefly, 1×10⁶ CRC cells were resuspended in 1× binding buffer, then 5 µL of Annexin V-FITC and 5 µL of PI reagent were added to the tube. The mixture was incubated in the dark at room temperature for 15 minutes, followed by analysis using a FACS Canto II flow cytometer (BD Biosciences, San Jose, CA,USA) within 1 h.

*Cell proliferation assays and In vitro IC50 assays*: For the CCK-8 assay, SW480 and SW620 cells transfected with shRNA or plasmids were seeded in 96-well plates at a density of 1 × 10³ cells per well. Cell proliferation was assessed using the Cell Counting Kit-8 (CCK-8) assay (Biosharp, Hefei, China). Cell viability was measured by recording the absorbance at 450 nm every 24 h for 5 consecutive days.

For the *in vitro* IC50 assay, cells were seeded into a 96-well plate at a density of 2 × 10³ cells per well, cultured for 24 h, and then treated with various concentrations of CPT-11 for 48 h. Cell viability was measured at 450 nm, and the IC50 value was calculated using GraphPad Prism 9.0 (GraphPad Software, La Jolla, CA, USA).

*Colony formation assay*: For the colony formation assay, shRNA or plasmid-transfected SW480 and SW620 cells were seeded into 6-well plates at a density of 1 × 10³ cells per well and incubated for 14 days. The cells were then fixed in 4% paraformaldehyde for 20 min and stained with 0.1% crystal violet for 15 min at room temperature. The visible colonies were counted and photographed.

*EdU assay*: The EdU assay was performed according to the instructions provided with the BeyoClick™ EdU-594 assay kit (Beyotime, Shanghai, China). CRC cells were cultured in 96-well plates at a density of 1 × 10³ cells per well for 48 h. After incubation with 10 μM EdU for 2 h, the cells were stained with fluorescent dyes, and the nuclei were stained with 1× Hoechst 33342 at room temperature for 10 min.

*Immunoﬂuorescence (IF) staining and Confocal microscopy analysis*: Cells were cultured in 48-well plates at a density of 1 × 10⁴ cells per well for 24 h and then fixed with 4% paraformaldehyde for 15 min, followed by permeabilization with 0.5% Triton X-100 for 15 min. The cells were then blocked with 5% BSA for 1 h and incubated with the primary antibody at 4°C overnight. The next day, the cells were incubated with appropriate fluorescence-conjugated secondary antibodies for 1 h at room temperature in the dark. The samples were stained with DAPI (Abiowell, Changsha, China), and images were acquired using an LSM 900 confocal microscope (Zeiss, Thornwood, NY, USA).

*Immunohistochemistry (IHC) and TUNEL assays*: The formalin-fixed paraffin-embedded CRC tissue was deparaffinized with 100% xylene and then rehydrated using ethanol at different concentrations. After inhibiting endogenous peroxidase activity, IHC staining was performed using the DAB Colorimetric Reagent Kit (ZSGB-BIO, Beijing, China). TUNEL staining was conducted using the TUNEL BrightRed Apoptosis Detection Kit (Vazyme, Nanjing, China) according to the manufacturer's instructions.

*RNA sequencing and Transcriptomic analysis*: For RNA-seq of CRC cells, we sorted SW620 cells treated with CPT-11 alone and cells treated with both PRMT5 inhibition and CPT-11. For RNA-seq of mouse tissues, tumors were collected from mice treated with either DMSO or a combination of PRMT5i and CPT-11. Total RNA was extracted using TRIzol reagent (Invitrogen, Carlsbad, CA, USA), followed by library preparation and RNA sequencing on the Illumina NovaSeq 6000 platform.

For the transcriptomes of CRC cells, We conducted GSVA and GSEA to analyze pathways involved in DNA damage and mismatch repair. For the transcriptomes of mouse tumors, intratumoral heterogeneity analysis was performed using the DEPTH2 R package. Immune infiltration was assessed using ESTIMATE and mMCP-counter, depending on whether mouse genes were converted to their human orthologs, as required by each algorithm.

*RNA isolation and qRT-PCR*: Total RNA was extracted using the TRIzol reagent (Invitrogen, Carlsbad, CA, USA). Subsequently, cDNA was synthesized using the Evo M-MLV RT Mix Kit (Accurate Biology, Changsha, China) and RNase-free water, following the manufacturer’s instructions. The cDNA was then amplified and analyzed by qRT-PCR using the SYBR Green Premix Pro Taq HS qPCR Kit (Accurate Biology, Changsha, China) on the QuantStudio™ 7 Real-Time PCR System (Thermo Fisher Scientific, Waltham, MA, USA). The beta-actin (β-actin) gene was used as an internal reference. The primer sequences are shown in Table S1.

*Dual-luciferase reporter assay*: The binding sites of PRMT5 and PMS2 promoter, or PMS2 promoter were cloned into the pGL3-Basic vector (IGE, Guangzhou, China). SW480 and SW620 cells were seeded in a 24-well plate and incubated for 24 h, followed by treatment with CPT-11 (50 μM) for an additional 24 h. Next, the PMS2 promoter-wt or PMS2 promoter-mut reporter construct and PRMT5 overexpression plasmid or a control vector were co-transfected using the Lipofectamine 3000 kit (Invitrogen, Carlsbad, CA, USA). Renilla luciferase was used for normalization. The luciferase activities of Renilla and firefly were measured after 48 h using the Dual-Luciferase Reporter Assay Kit (Promega, Madison, WI, USA).

*Ex vivo DCs activation and T cell response*: Spleens from Balb/c mice were harvested and prepared into single-cell suspensions. The cells were incubated with anti-CD11c microbeads (Miltenyi, Bergisch Gladbach, Germany) and anti-CD8a microbeads (Miltenyi, Bergisch Gladbach, Germany) for 30 min, respectively. The labeled cells were then separated using a MACS LS Column on a MACS separator (Miltenyi, Bergisch Gladbach, Germany). CD11c^+^ dendritic cells (DCs) and CD8^+^ T cells were retained on the column and eluted after removal from the magnetic field. Subsequently, the microbead-isolated cells were co-cultured with tumor cells from different intervention groups, and the activation markers of CD11c^+^ DCs and CD8^+^ T cells were detected by flow cytometry, followed by calculating the proportion of activated cells.

*Flow cytometric analysis in tumor-bearing mice*: Tumor tissues were minced into small pieces and then digested using a 10X triple digestive enzyme mixture containing DNase (200 mg/ml, Sigma-Aldrich, St Louis, MO, USA), hyaluronidase (1 mg/ml, Sigma-Aldrich, St Louis, MO, USA), and collagenase (10 mg/ml, Sigma-Aldrich, St Louis, MO, USA) for 30 min at 37°C. After incubation, the cell suspension was filtered through a 70 μm strainer to remove undigested tissue and obtain a single-cell suspension. Cells were then blocked for Fc receptors using a CD16/CD32 antibody and dead cells were excluded by zombie staining. The cells were subsequently stained with a panel of fluorochrome-conjugated antibodies targeting specific immune markers, including CD3, CD45, CD11c, CD8a, and various cytokines. Flow cytometry was performed to analyze the expression of these immune molecules using a FACS Canto II flow cytometer (BD Biosciences, San Jose, CA, USA), and the results were analyzed using FlowJo software (BD Biosciences, San Jose, CA, USA). Detailed information on the antibodies used in the experiments is provided in Table S3.

*Measurement of biochemical variables*: Serum extraction was performed via centrifugation. AST, ALT, CRE, and BUN levels in the serum of all mice were measured using commercial assay kits (Njjcnio, Nanjing, China) according to the manufacturer's protocols.


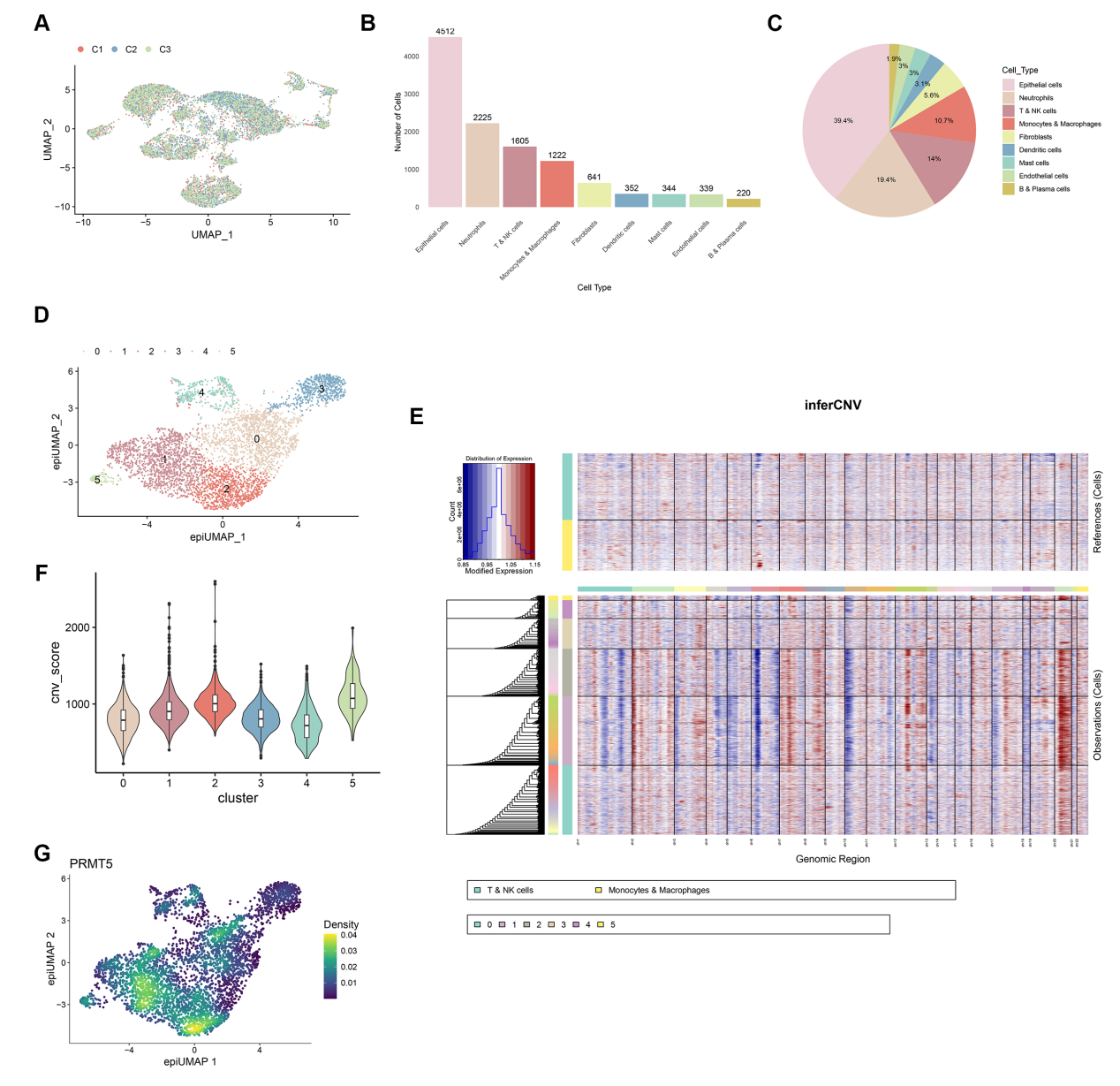


**Figure S1. PRMT5 exerts DDR-related effects in MSS CRC.**

1. UMAP plots of single cells identified by scRNA-seq, color-coded by CRC tissues. B, C) Bar graph (B) and pie chart (C) showing the relative numbers and proportions of different cell types, color-coded by relevant cell type. D) UMAP plot of epithelial cells, with different color codes representing various subclusters. E) Hierarchical heatmap showing copy number variations (CNVs) in epithelial cells. F) Violin plots displaying the distribution of CNV scores across different epithelial cell subclusters. G) Density plot showing the expression distribution of PRMT5 in epithelial cells.

**
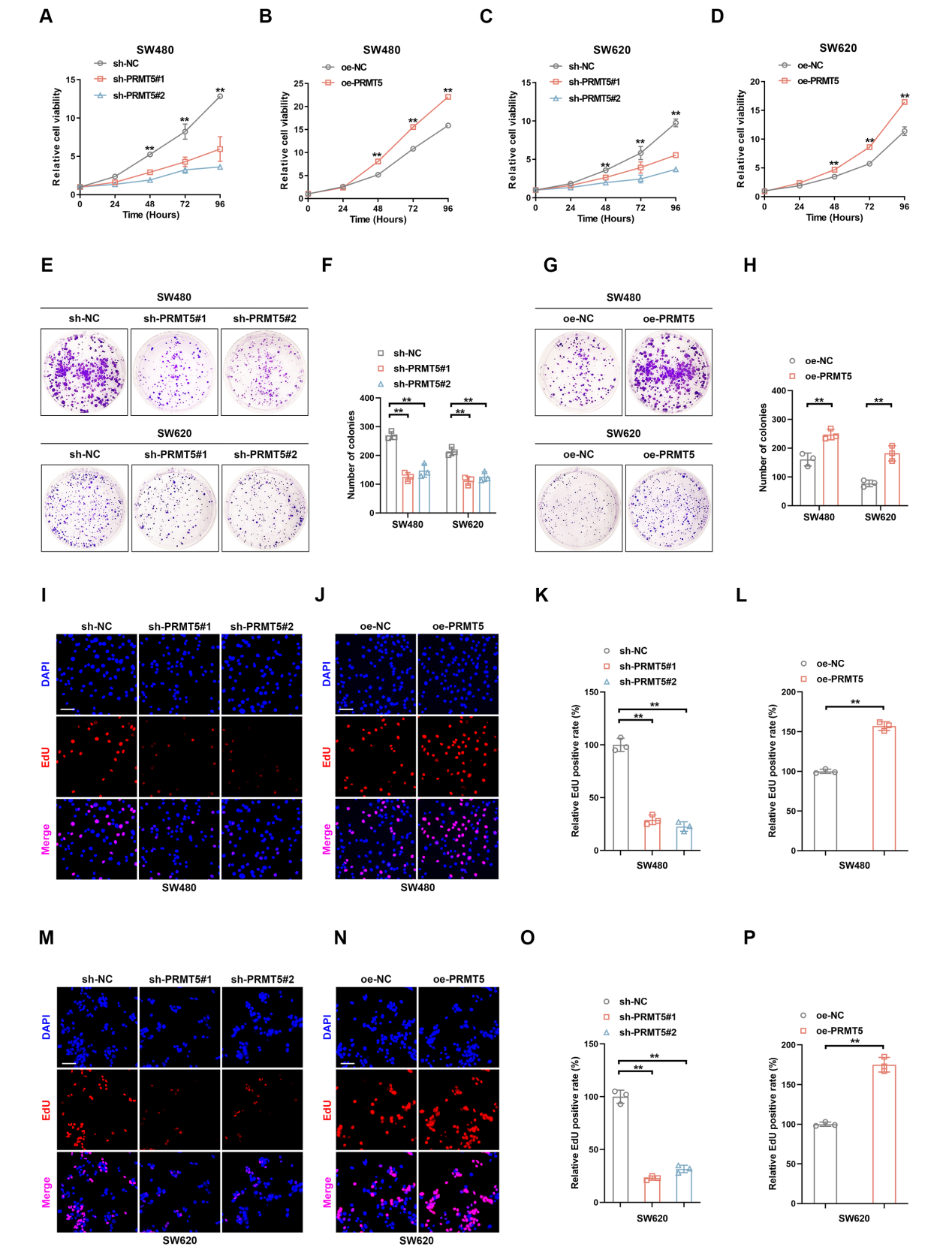
**

**Figure S2. PRMT5 facilitate the viability and DNA replication in MSS CRC cells**

A-P) The proliferative ability of SW480 and SW620 cells with PRMT5 knockdown or overexpression was assessed using the CCK-8 assay (A-D), colony formation assay (E-H), and EdU assay (I-P). The p-values were calculated using two-way ANOVA (A-D), one-way ANOVA (F, K, O) and two-tailed Student's t-test (H, L, P). Scale bar = 20 μm. Error bars show the mean ± SD. ns, p > 0.05, **p* < 0.05, ***p* < 0.01.


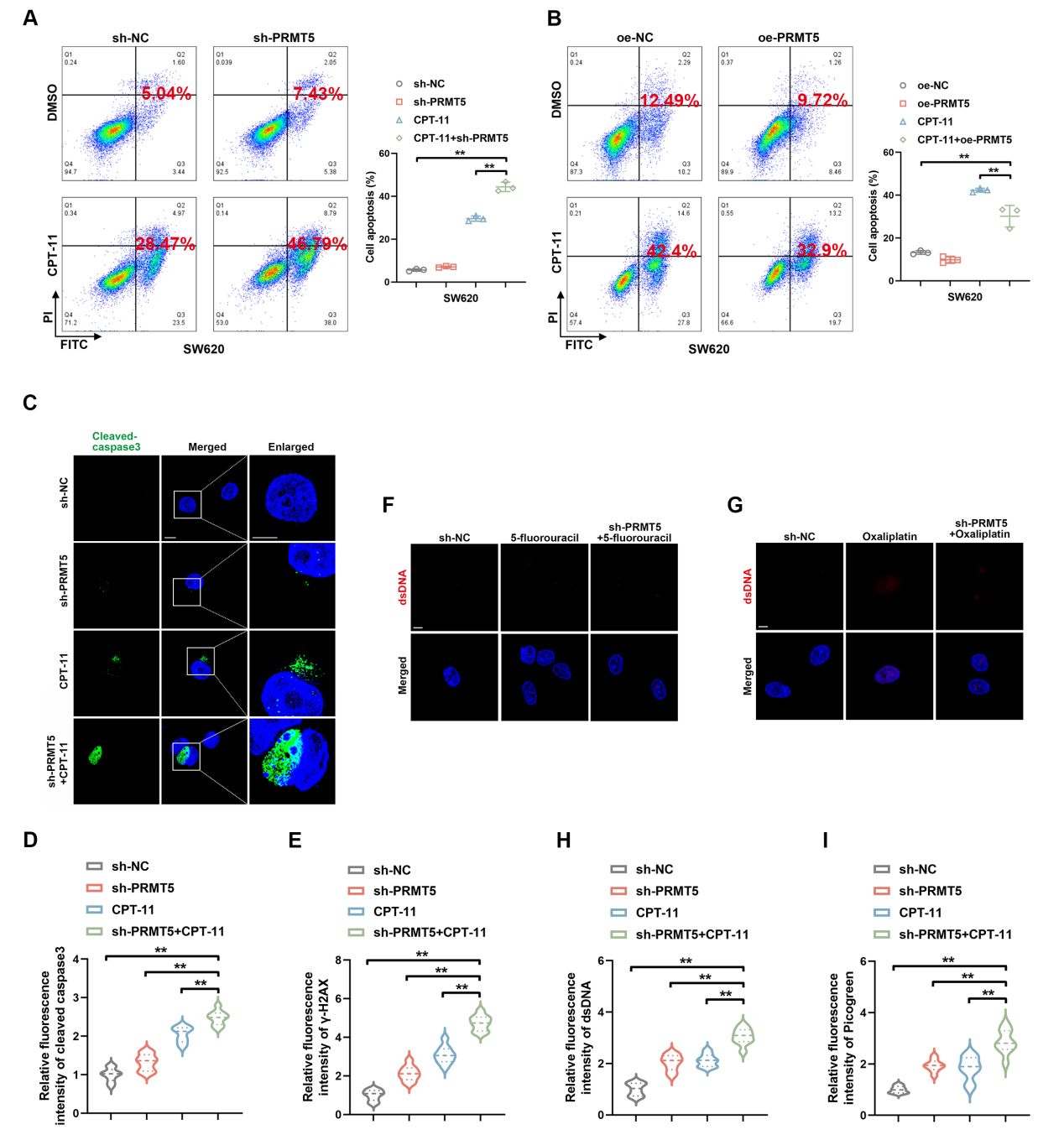


**Figure S3. PRMT5 inhibition and CPT-11 synergistically inhibit proliferation and promote apoptosis in MSS CRC.**

A, B) Flow cytometry analysis of apoptosis in PRMT5 knockdown (A), PRMT5 overexpression (B) and paired control SW620 cells treated with DMSO or CPT-11 (50 μM) for 48 h. The p-values were calculated using one-way ANOVA. C, D) Representative fluorescence images (C) and quantitative fluorescence intensity analysis (D) showing the cellular localization and expression of cleaved caspase-3 in PRMT5 knockdown and paired control CRC cells, with or without CPT-11 (50 μM) treatment. Scale bar = 5 μm. Fluorescence intensity normalized to control. The p-values were calculated using one-way ANOVA. E) Quantitative fluorescence intensity analysis of γ-H2AX in PRMT5 knockdown and paired control CRC cells, with or without CPT-11 (50 μM) treatment. Fluorescence intensity normalized to control. The p-values were calculated using one-way ANOVA. F, G) Representative fluorescence images showing cellular localization and expression of dsDNA in PRMT5 knockdown and paired control CRC cells, with or without 5-fluorouracil(50 μg/ml) (F) or oxaliplatin (50 μM) (G) treatment. Scale bar = 5 μm. H, I) Quantitative fluorescence intensity analysis of dsDNA (H), and Picogreen (I) in PRMT5 knockdown and paired control CRC cells, with or without CPT-11 (50 μM) treatment. Fluorescence intensity normalized to control. The p-values were calculated using one-way ANOVA. Error bars show the mean ± SD. ns, p > 0.05, **p* < 0.05, ***p* < 0.01.


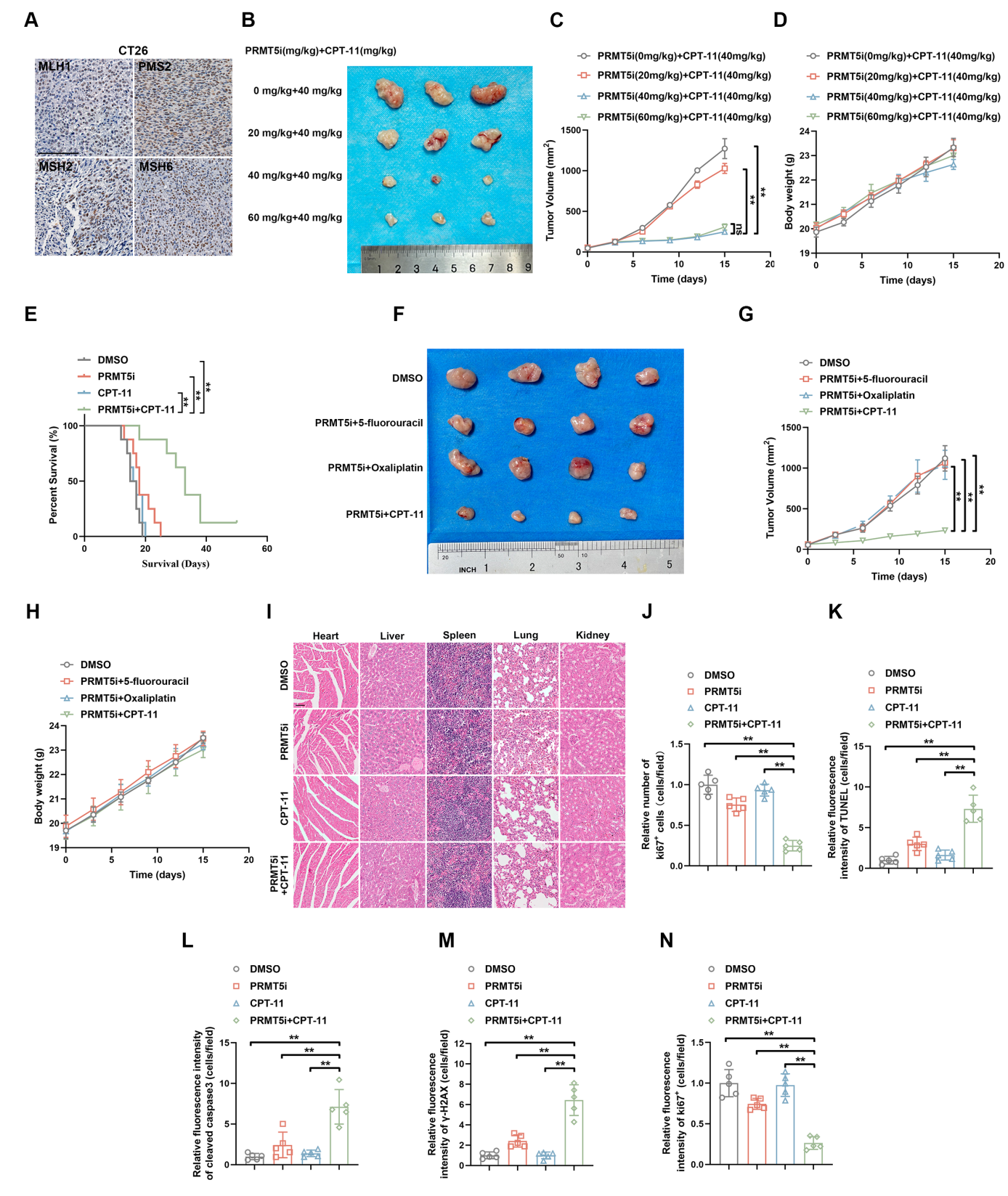


**Figure S4. The combination of PRMT5i and CPT-11 exhibits synergistic anti-tumor effects in vivo.**

1. IHC staining showing MLH1, PMS2, MSH2, MSH6 protein levels in CT26 tumor tissues. Scale bar = 50 μm. B-D) Representative tumor images (B), tumor growth curves (C), body weight (D) of mice treated with DMSO, and dose-escalating PRMT5i + CPT-11 (n = 3). The p-values were calculated using two-way ANOVA (C). E) Kaplan-Meier survival curves in all groups (n = 8). The p-values were calculated using Log-rank test. F-H) Representative tumor images (F), tumor growth curves (G), body weight (H) of mice treated with DMSO, and PRMT5i (40 mg/kg) + 5-fluorouracil (40 mg/kg), PRMT5i (40 mg/kg) + oxaliplatin (2.5 mg/kg) and PRMT5i (40 mg/kg) + CPT-11 (40 mg/kg) (n = 4). The p-values were calculated using two-way ANOVA (G). I) Representative images of organ indexes, including heart, liver, spleen, lungs, and kidneys, in subcutaneous mice. Scale bar = 50 μm. J, K) Quantification of positive ki-67 cells (J) and TUNEL mean fluorescence intensity (K) in the indicated groups. Fluorescence intensity normalized to control. The p-values were calculated using one-way ANOVA. L-N) Quantitative fluorescence intensity analysis of cleaved caspase-3 (L), γ-H2AX (M), and ki-67 (N) in the indicated tumor tissues, with fluorescence intensity normalized to control. The p-values were calculated using one-way ANOVA. Error bars show the mean ± SD. ns, p > 0.05, **p* < 0.05, ***p* < 0.01.

**
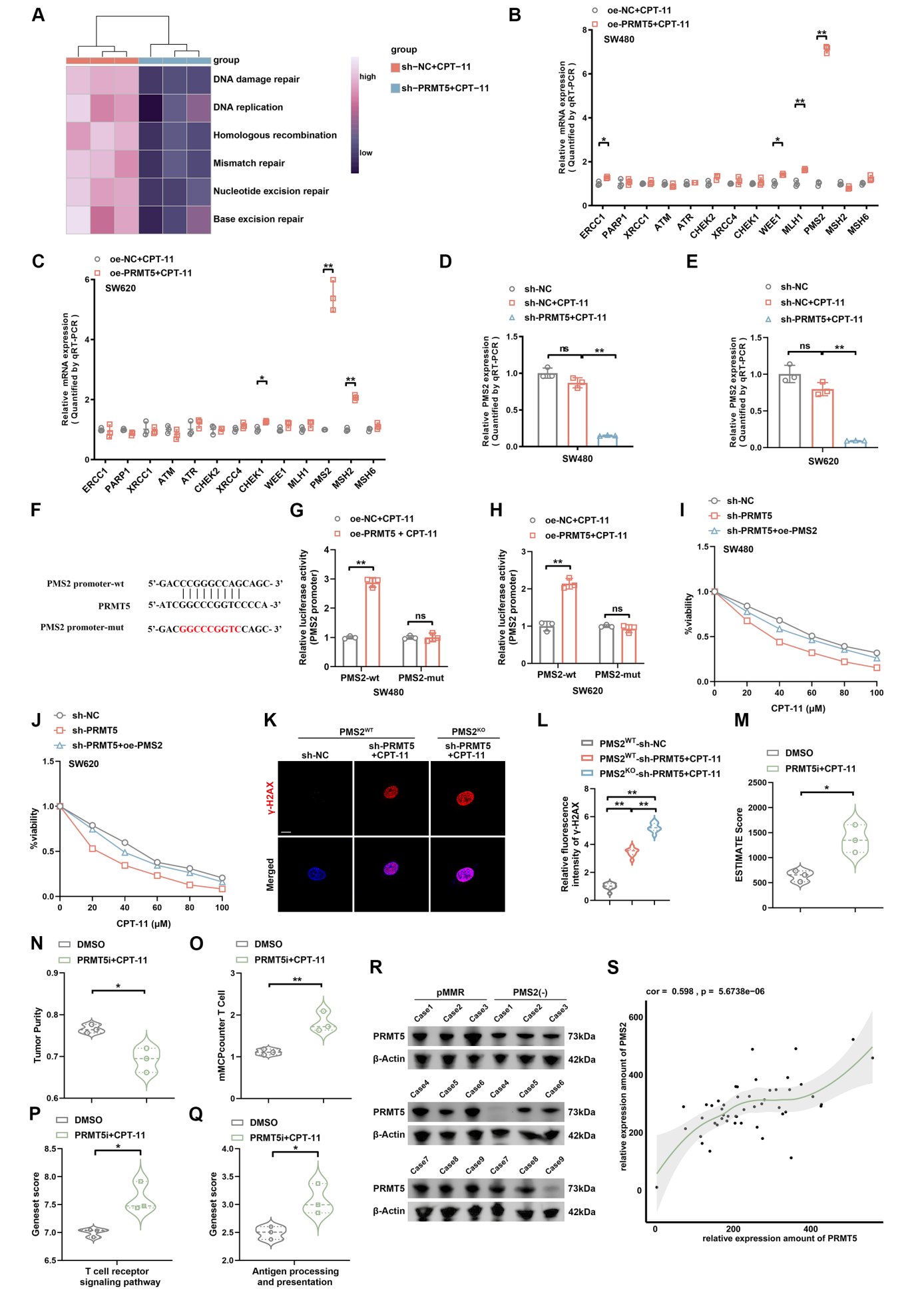
**

**Figure S5. The combination of PRMT5 inhibition and CPT-11 induces a state resembling dMMR in MSS CRC.**

1. Heatmap showing the response of multiple DNA repair pathways in SW620 cells comparing CPT-11 vs. PRMT5 inhibition combined with CPT-11, analyzed by GSVA. B, C) qRT-PCR analysis of the expression of 13 candidate DDR genes in control and PRMT5-overexpressing SW480 (B) and SW620 (C) cells following CPT-11 treatment. The p-values were calculated using a two-tailed Student's t-test. D, E) qRT-PCR analysis of the expression of PMS2 in control, CPT-11, PRMT5-silencing + CPT-11 SW480 (D) and SW620 (E) cells. The p-values were calculated using a one-way ANOVA. F) Schematic illustrating the sequence alignment of PRMT5 with the promoter of PSM2. G, H) The luciferase activities of the PMS2 promoter-wt plasmid or PMS2 promoter-mut plasmid quantified following transfection with the PRMT5-overexpressing plasmid in CPT-11-treated SW480 (G) and SW620 (H) cells. The p-values were calculated using a two-tailed Student's t-test. I, J) Cell viability of SW480 (I) and SW620 (J) cells was assessed using the CCK-8 assay after 48 h of treatment with increasing concentrations of CPT-11 across different groups. K, L) Representative fluorescence images (K) and quantitative analysis (L) showing cellular localization and expression of γ-H2AX in indicated group. Fluorescence intensity normalized to control. Scale bar = 5 μm. The p-values were calculated using a one-way ANOVA. M, N) ESTIMATE score of the indicated treatment groups (n=3). The p-values were calculated using a two-tailed Student's t-test. O) mMCPCounter analysis of T cell infiltration in the indicated treatment groups (n=3). The p-values were calculated using a two-tailed Student's t-test. P, Q) Gene set scores analysis of T cell receptor signaling (P) and antigen presentation pathway (Q) in the indicated treatment groups (n=3). The p-values were calculated using a two-tailed Student's t-test. R) Western blot analysis of PRMT5 levels in pMMR CRC tissues and PMS2-deficient CRC tissues (n=9). S) Correlation analysis of PRMT5 and PMS2 level in MSS CRC patients (n = 49). Error bars show the mean ± SD. ns, p > 0.05, **p* < 0.05, ***p* < 0.01.

**
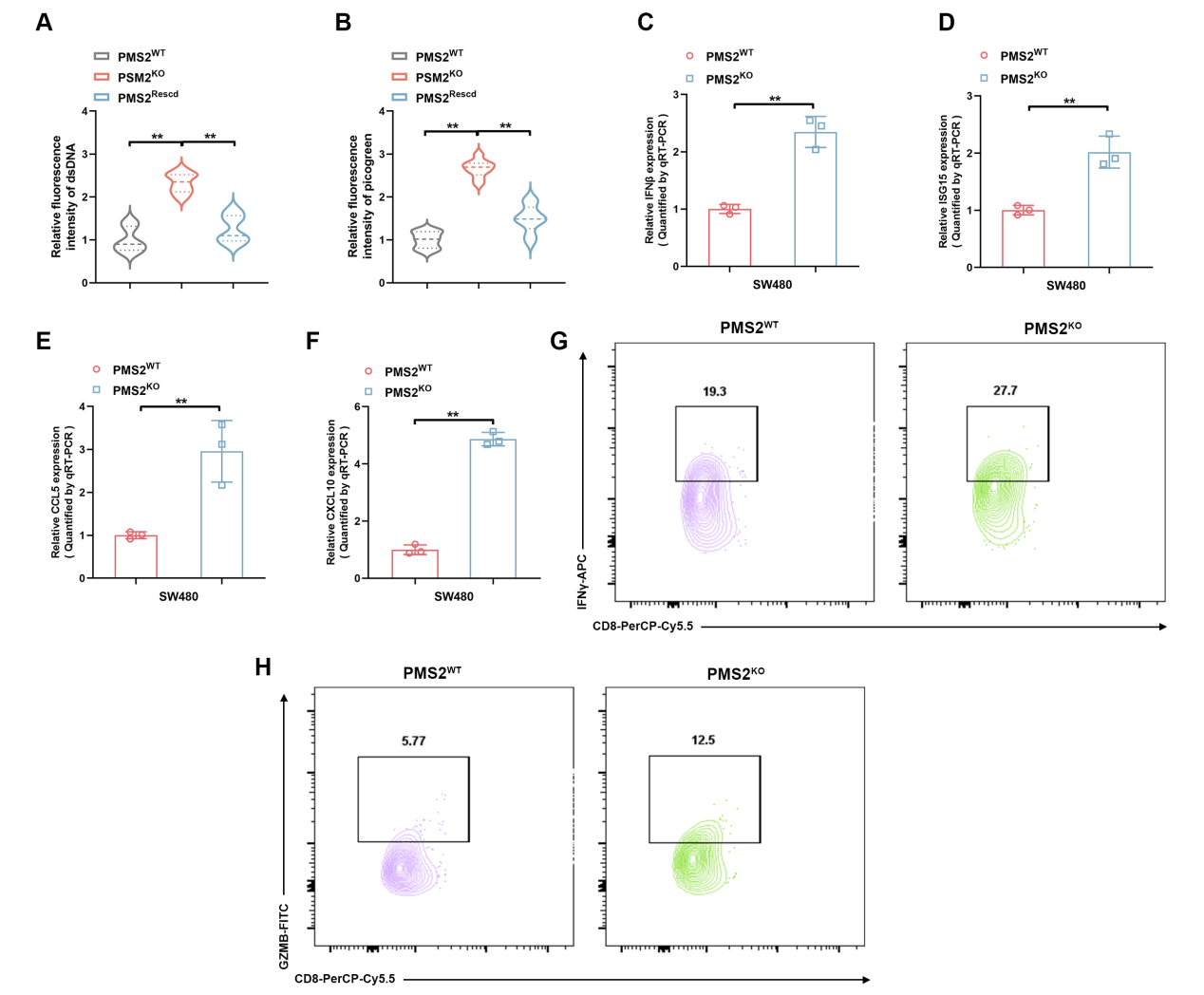
**

**Figure S6. PMS2 deficiency facilitate the activation of cGAS/STING pathway.**

A, B) Quantitative fluorescence intensity analysis of dsDNA (A) and Picogreen (B) in PMS2^WT^, PMS2^KO^ and PMS2^rescued^ CRC cells. Fluorescence intensity normalized to control. The p-values were calculated using one-way ANOVA. C-F) qRT-PCR analysis of the effect of PMS2 knockout on the expression of IFN-β (C), ISG15 (D), CCL5 (E), and CXCL10 (F) in SW480 cells. The p-values were calculated using a two-tailed Student's t-test. G, H) Representative flow cytometry images showing IFNγ^+^ (G) and GZMB^+^ (H) expression on CD8^+^ T cells in the PMS2^WT^ and PMS2^KO^ groups (n = 3). Error bars show the mean ± SD. ns, p > 0.05, **p* < 0.05, ***p* < 0.01.


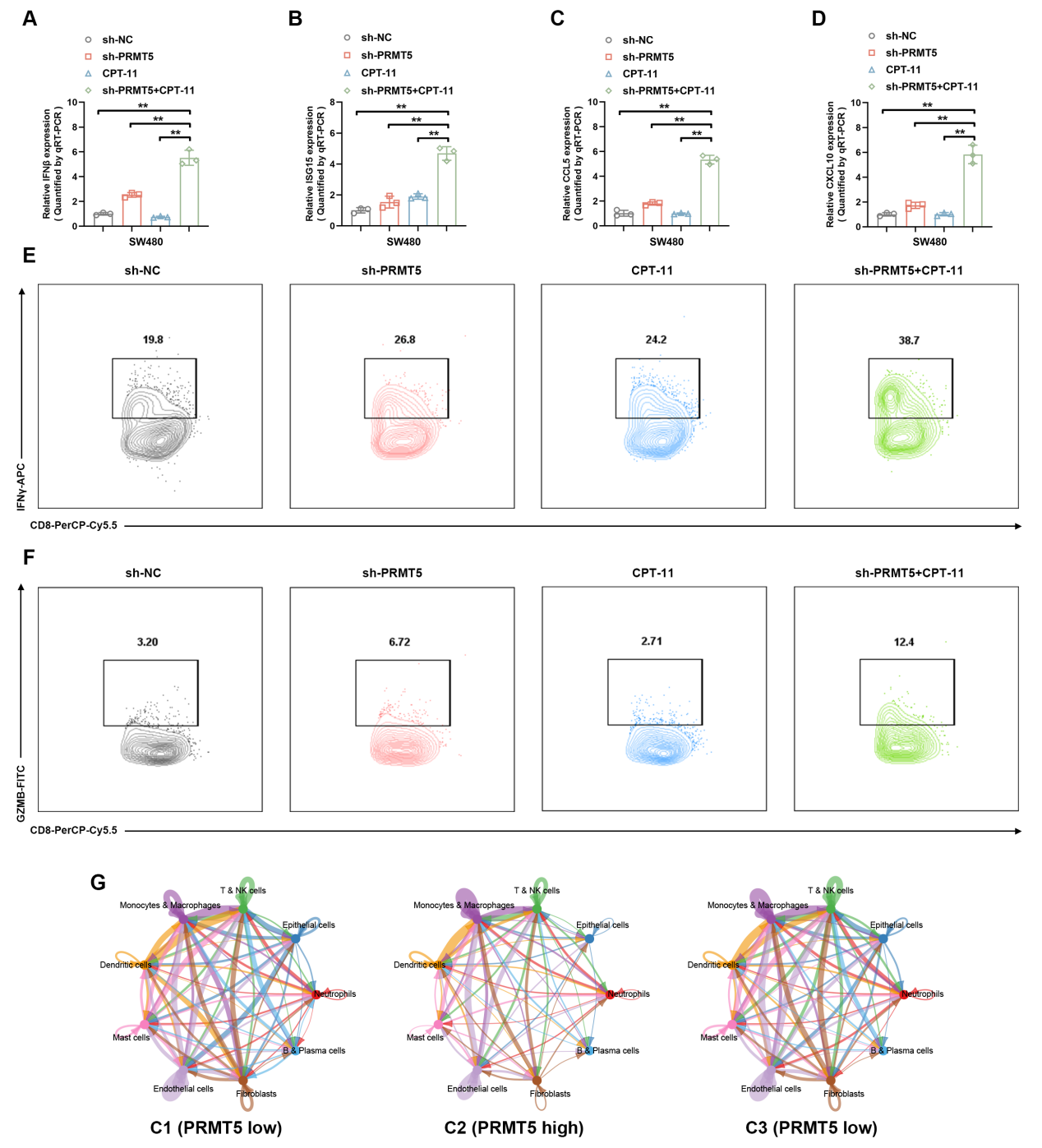


**Figure S7. PRMT5 silencing in combination with CPT-11 promotes T cell anti-tumor immunity.**

A-D) qRT-PCR analysis of IFN-β (A), ISG15 (B), CCL5 (C), and CXCL10 (D) levels in SW480 cells across different treatment groups: control, PRMT5 silencing, CPT-11, and PRMT5 silencing + CPT-11. The p-values were calculated using one-way ANOVA. E, F) Representative flow cytometry images showing IFNγ^+^ (E) and GZMB^+^ (F) expression on CD8^+^ T cells in all groups (n = 3). G) CellChat analysis of scRNA-seq data in MSS patients. (n = 3). Error bars show the mean ± SD. ns, p > 0.05, **p* < 0.05, ***p* < 0.01.

**
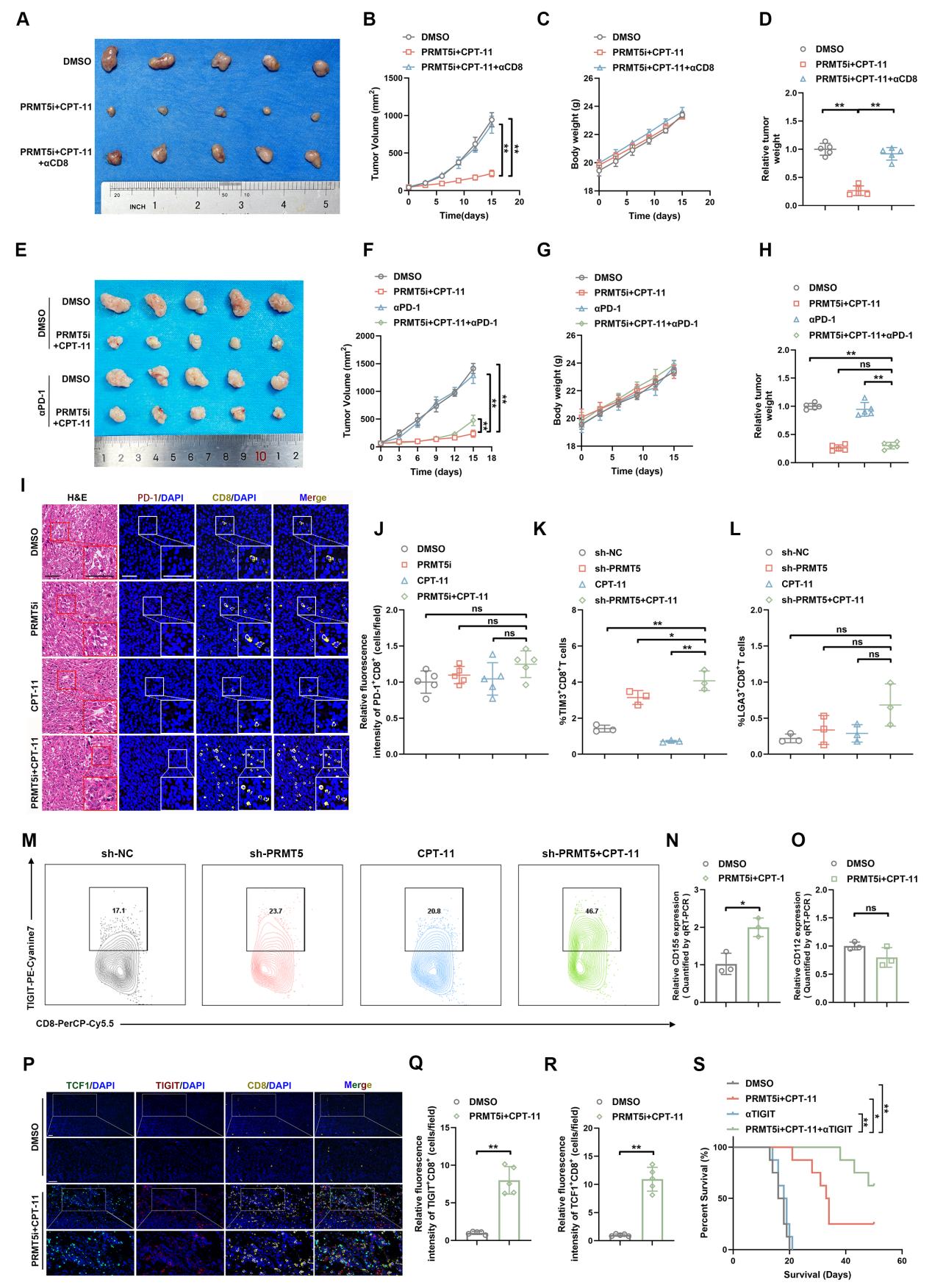
**

**Figure S8. Dual-drug-induced dMMR escapes anti-PD1 therapy but is susceptible to anti-TIGIT treatment.**

A-D) Representative images (A), tumor growth curves (B), body weight (C), and relative tumor weight (D) of tumor-bearing mice treated with DMSO, PRMT5i (40 mg/kg) + CPT-11 (40 mg/kg), or PRMT5i + CPT-11 + αCD8 (100 µg per mouse) (n = 5). The p-values were calculated using two-way ANOVA (B) and one-way ANOVA (D). E-H) Representative images (E), tumor growth curves (F), body weight (G), and relative tumor weight (H) of tumor-bearing mice treated with DMSO, PRMT5i (40 mg/kg) + CPT-11 (40 mg/kg), αPD-1 (100 µg per mouse), or PRMT5i + CPT-11 + αPD-1 (n = 5). The p-values were calculated using two-way ANOVA (F) and one-way ANOVA (H). I, J) Representative fluorescence images (I) and quantitative analysis (J) of PD-1 levels on CD8^+^ cells in the indicated mouse tissues. Fluorescence intensity normalized to control. The p-values were calculated using one-way ANOVA. Scale bar = 50 μm. K, L) Expression levels of TIM3 (G) and LAG3 (H) on CD8^+^ T cells isolated from the spleens of mice in the indicated groups (n = 3). The p-values were calculated using one-way ANOVA. M) Representative flow cytometry images showing TIGIT^+^ expression on CD8^+^ T cells isolated from the spleens of mice in the indicated groups (n = 3). N, O) qRT-PCR analysis of CD155 (N) and CD112 (O) levels in mouse tissues across different treatment groups: DMSO, PRMT5i + CPT-11. The p-values were calculated using two-tailed Student's t-test. P-R) Representative fluorescence images (P) and quantitative analysis of TIGIT (Q) or TCF1 (R) levels on CD8^+^ cells in the indicated mouse tissues. Fluorescence intensity normalized to control. The p-values were calculated using two-tailed Student's t-test. Scale bar = 50 μm. S) Kaplan-Meier survival curves in indicated groups (n = 8). The p-values were calculated using Log-rank test. Error bars show the mean ± SD. ns, p > 0.05, **p* < 0.05, ***p* < 0.01.

**Table S1. qRT-PCR Primer used in the experiments.**

| **Gene** | **Sequence (5’-3’)** | **Application** |
| --- | --- | --- |
| PRMT5-human | F: CGTCCTCCACCTAATGCCTATGAAC  R: CTCCTCTTCTGGTACTCGGTCTAGC | qRT-PCR |
| β-actin-human | F: CCTGGCACCCAGCACAAT  R: GGGCCGGACTCGTCATAC | qRT-PCR |
| ERCC1-human | F: CAGCAAGGAAGAAATTTGTGATACCC  R: GTGAGATGGCATATTCGGCGTAG | qRT-PCR |
| PARP1-human | F: CAGAGTATGCCAAGTCCAACAGAAG  R: CAGCGGTCAATCATGCCTAGC | qRT-PCR |
| XRCC1-human | F: CCGTGTGAAGGAGGAGGATGAG  R: GCACTAGAAGCCTGGAGGGTAG | qRT-PCR |
| ATM-human | F: AGAGATTGTGGTGGAGTTATTGATGAC  R: ATGAGGTGGATTAGGAGCAGGATC | qRT-PCR |
| ATR-human | F: ACCAGTGAAAGGGCATTCCAAAG  R: TAGTCGCTGCTCAATGTCAAGAAC | qRT-PCR |
| CHEK2-human | F: AACTCCAGCCAGTCCTCTCAC  R: GGTTCTTGGTCCTCAGGTTCTTG | qRT-PCR |
| XRCC4-human | F: AGAAGCTGATGACATGGCAATGG  R: GGTCCTGCTCCTGACAACAATG | qRT-PCR |
| CHEK1-human | F: GAAGGTGCCTATGGAGAAGTTCAAC  R: TCATATCTACAATCTTCACTGCGACTG | qRT-PCR |
| WEE1-human | F: GTATGTGCTGCTGGTGCTGAAC  R: CACTTGTGGTATCCGAGGTAATCTAC | qRT-PCR |
| MLH1-human | F: GTTCGTGGCAGGGGTTATTC  R: CCAAAGCCTCACCTCGAAAG | qRT-PCR |
| PMS2-human | F: GGGCTGCCATTCAAACCAGG  R: GAAGCTGACATGTCCTGAGT | qRT-PCR |
| MSH2-human | F: AGTTTCATCACTGTCTGCGG  R: AGGCTGCTTAATCCACTGGT | qRT-PCR |
| MSH6-human | F: CGCCATCCTTGCATTACGAA  R: ACTTCAGCAGGGACGTAACA | qRT-PCR |
| CXCL10-human | F: CTCCAGTCTCAGCACCATGA  R: GCTCCCCTCTGGTTTTAAGG | qRT-PCR |
| CCL5-human | F: CTCGCTGTCATCCTCATTGCTACTG  R: TGGACGACTGCTGGGTTGGAG | qRT-PCR |
| IFNβ-human | F: CTTGGATTCCTACAAAGAAGCAGC  R: TCCTCCTTCTGGAACTGCTGCA | qRT-PCR |
| ISG15-human | F: CAGAAGATCGGCGTGCAC  R: GCTCAGAGGTTCGTCGCATT | qRT-PCR |
| PMS2 P1-human | F: CAGGCTCTTTGACGTCACGAA  R: CATTGGCTGCTTTCGACGTTG | qRT-PCR |
| PMS2 P2-human | F: AGTCTGAGTGAGTCCCTGGC  R: TTCTGCCATGCCGATGTACC | qRT-PCR |
| PMS2 P3-human | F: CAATTGATCCTCCCGCCTCAGT  R: AAAATACAGAAATTGGCCGGGTGTG | qRT-PCR |
| PMS2 P4-human | F: AAGTGGAAAGAATGGAGTGGTGAC  R: AGACCACTCTCAGCAACATAGC | qRT-PCR |
| PMS2 P5-human | F: ATGAAGACTATTGTTTAGGGGCCGG  R: GTGCAGTGGTGTGATTTTAGCTCA | qRT-PCR |
| PRMT5-mouse | F: TGAAGCGGCTATGTTACAGGAGTTG  R: AGTGTGGATGTGGTTGGTCAGAAC | qRT-PCR |
| β-actin-mouse | F: GTGCTATGTTGCTCTAGACTTCG  R: ATGCCACAGGATTCCATACC | qRT-PCR |
| CXCL10-mouse | F: AATGAGGGCCATAGGGAAGC  R: AGCCATCCACTGGGTAAAGG | qRT-PCR |
| CCL5-mouse | F: TGCAGAGGACTCTGAGACAGC  R: GAGTGGTGTCCGAGCCATA | qRT-PCR |
| IFNβ-mouse | F: TGGGTGGAATGAGACTATTGTTGTAC  R: AGTGGAGAGCAGTTGAGGACATC | qRT-PCR |
| ISG15-mouse | F: GAACAAGTCCACGAAGACCAG  R: GCAGCTCCTTGTCCTCCAT | qRT-PCR |
| CD155-mouse | F: TCATCATCATCGCAGCACTATACAC  R: AGTCTACAGTCGCCGTTCACAG | qRT-PCR |
| CD112-mouse | F: GAACATTGCTGGTGCTACTCCTG  R: TCGCCATCATTTCCTCCTCCTC | qRT-PCR |

**Table S2. Plasmid sequences used in the experiments.**

| **Gene** | **Sequence** | **Application** |
| --- | --- | --- |
| sh-PRMT5#1  -human | GCCCAGTTTGAGATGCCTTAT | sh-RNA |
| sh-PRMT5#2  -human | GCCATCTATAAATGTCTGCTA | sh-RNA |
| sh-PRMT5  -mouse | CCCATCAAATACTCTCAATAT | sh-RNA |
| sg-PMS2  -human | TTCATGCTGAGCGACAGCCC | knockout-RNA |
| sg-PMS2  -mouse | TGCAAGCGTTGGGACTCGAC | knockout-RNA |

**Table S3. Antibodies used in the experiments.**

| **Product** | **Source** | **No. of Catalogue** |
| --- | --- | --- |
| **Primary antibody:** |  |  |
| ***Western blot:*** |  |  |
| β-actin | Proteintech | 66009-1-Ig |
| anti-PRMT5 | AiFang biological | AF300453 |
| anti-PMS2 | Proteintech | 66075-1-Ig |
| anti-γ-H2AX | Sigma-Aldrich | 05-636 |
| anti-cleaved caspase3 | Cell Signaling Technology | 9661S |
| anti-TBK1 | Cell Signaling Technology | 38066S |
| anti-IRF3 | AiFang biological | AF300775 |
| anti-STING | Cell Signaling Technology | 13647S |
| anti-p-TBK1 | Cell Signaling Technology | 5483S |
| anti-p-IRF3 | Cell Signaling Technology | 29047S |
| anti--human-p-STING | Cell Signaling Technology | 50907S |
| anti-mouse-p-STING | Cell Signaling Technology | 72971S |
| ***IHC:*** |  |  |
| anti-PRMT5 | AiFang biological | AF300453 |
| anti-PSM2 | Immunoway | YT3804 |
| anti-ki67 | Proteintech | 28074-1-AP |
| anti-MLH1 | Abiowell | AWA13135 |
| anti-MSH2 | Abiowell | AWA04303 |
| anti-MSH6 | Abiowell | AWA13239 |
| ***IF and mIF*** |  |  |
| anti-γ-H2AX | Cell Signaling Technology | 9718T |
| anti-cleaved caspase3 | Cell Signaling Technology | 9661S |
| anti-dsDNA | NOVUS | NBP3-07670 |
| anti-ki67 | Proteintech | 28074-1-AP |
| anti-mouse-CD4 | Abcam | ab183685 |
| anti-mouse-CD8 | Abcam | ab217344 |
| anti-mouse-CD11c | Abcam | ab219799 |
| anti-mouse-TIGIT | Abcam | ab300073 |
| anti-mouse-PD-1 | Cell Signaling Technology | 84651T |
| anti-mouse-TCF1 | Cell Signaling Technology | 2203T |
| anti-human-CD4 | Cell Signaling Technology | 93518S |
| anti-human-CD8 | Cell Signaling Technology | 85336S |
| anti-human-CD11c | Abcam | ab52632 |
| ***CHIP*** |  |  |
| anti-PRMT5 | Invitrogen | MA1-25470 |
| anti-H3R2me2s | Invitrogen | PA5-116966 |
| anti-IgG | Absin | abs20038 |
| ***Flow cytometry*** |  |  |
| CD16/CD32 | Invitrogen | 14-0161-82 |
| ***Panel 1：*** |  |  |
| anti-mouse-CD11c | BioLegend | 117329 |
| anti-mouse-CD80 | BioLegend | 104733 |
| anti-mouse-CD86 | BioLegend | 105011 |
| anti-mouse-IA/IE | BioLegend | 107605 |
| ***Panel 2：*** |  |  |
| anti-mouse-CD8a | BioLegend | 100734 |
| anti-mouse-IFN-γ | Invitrogen | 17-7311-82 |
| anti-mouse-GZMB | BioLegend | 396404 |
| ***Panel 3:*** |  |  |
| anti-mouse-CD8a | BioLegend | 100734 |
| anti-mouse-TIGIT | BioLegend | 142108 |
| anti-mouse-TIM3 | Invitrogen | 13-5871-82 |
| anti-mouse-LAG3 | Invitrogen | 48-2231-82 |
| ***Panel 4:*** |  |  |
| Zoobie | BioLegend | 423101 |
| anti-mouse-CD45 | BioLegend | 103125 |
| anti-mouse-CD3 | BioLegend | 100222 |
| anti-mouse-CD4 | BioLegend | 100408 |
| anti-mouse-CD8a | BioLegend | 100734 |
| ***Panel 5:*** |  |  |
| Zoobie | BioLegend | 423101 |
| anti-mouse-CD45 | BioLegend | 103125 |
| anti-mouse-CD3 | BioLegend | 100222 |
| anti-mouse-CD8a | BioLegend | 100734 |
| anti-mouse-IFN-γ | Invitrogen | 17-7311-82 |
| anti-mouse-GZMB | BioLegend | 396404 |
| ***Panel 6:*** |  |  |
| Zoobie | BioLegend | 423101 |
| anti-mouse-CD45 | BioLegend | 103132 |
| anti-mouse-CD11c | BioLegend | 117329 |
| anti-mouse-CD80 | BioLegend | 104733 |
| anti-mouse-CD86 | BioLegend | 105011 |
| anti-mouse-IA/IE | BioLegend | 107605 |
| **Secondary antibody:** |  |  |
| ***Western blot:*** |  |  |
| anti-rabbit IgG-HRP | Proteintech | SA00001-2 |
| anti-mouse IgG-HRP | Proteintech | SA00001-1 |
| ***IF:*** |  |  |
| anti-rabbit ®488 | Abcam | ab150077 |
| anti-rabbit ®594 | Abcam | ab150080 |
| anti-mouse ®488 | Abcam | ab150113 |
| anti-mouse ®594 | Abcam | ab150116 |
